# Panose prevents acute-on-chronic liver failure by reducing bacterial infection in mice

**DOI:** 10.1172/JCI184653

**Published:** 2025-06-03

**Authors:** Jiaxin Li, Shihao Xie, Meiling Chen, Changze Hong, Yuqi Chen, Fengyuan Lyu, Niexin Tang, Tianqi Chen, Lingyan Zhao, Weihao Zou, Hongjuan Peng, Jingna Bao, Peng Gu, Bernd Schnabl, Jinjun Chen, Peng Chen

**Affiliations:** 1 Department of Pathophysiology, Guangdong Provincial Key Laboratory of Proteomics, School of Basic Medical Sciences, Southern Medical University, Guangzhou, China.; 2 Hepatology Unit, Department of Infectious Diseases, Nanfang Hospital, Southern Medical University, Guangzhou, China.; 3 Department of Pathogen Biology, Guangdong Provincial Key Laboratory of Tropical Diseases Research, School of Public Health, Southern Medical University, Guangzhou City, Guangdong Province, China.; 4 Key Laboratory of Infectious Diseases Research in South China (Ministry of Education), Southern Medical University, Guangzhou, Guangdong, China.; 5 Department of Critical Care Medicine, Nanfang Hospital, Southern Medical University, Guangzhou, China.; 6 Department of Medicine, UCSD, La Jolla, San Diego, California, USA.; 7 Department of Medicine, VA San Diego Health Care System, San Diego, California, USA.; 8 Hepatology Unit, Zengcheng Branch, Nanfang Hospital, Southern Medical University, Guangzhou, China.; 9 Guangdong Provincial Key Laboratory of Viral Hepatitis Research, China.

**Keywords:** Hepatology, Metabolism, Microbiology, Bacterial infections, Tight junctions

## Abstract

Acute-on-chronic liver failure (ACLF) is a leading cause of global liver-related mortality. Bacterial infection, especially in patients with decompensated cirrhosis, commonly triggers ACLF and is difficult to treat with antibiotics. Therefore, finding alternative strategies for preventing and managing bacterial infection is an urgent priority. Here, we observed that patients with bacterial infection and decompensated cirrhosis, as well as ACLF mice, exhibited lower fecal panose levels than uninfected controls. *Megamonas funiformis*, with 4α-glucanosyltransferase (4αGT) as a key enzyme for panose production, was identified as a potential panose producer. Animal experiments demonstrated that panose efficiently reduced liver injury and extended survival in ACLF mice by mitigating bacterial infection. Further results revealed that panose enhanced resistance to bacterial infection by inhibiting oxidative stress–induced gut barrier disruption, thereby limiting bacterial dissemination. Mechanistically, panose interacted with the solute carrier family 7 member 11 (SLC7A11, also known as xCT) protein to boost antioxidant glutathione levels in intestinal epithelial cells. These findings highlight panose’s potential in preventing bacterial infection, offering a valuable insight into mitigating ACLF progression.

## Abstract 2

Panose, a gut microbiota-derived prebiotic, reduces the risk of bacterial infection during acute-on-chronic liver failure by repairing oxidative stress-induced intestinal barrier dysfunction in mice.

## Graphical abstract

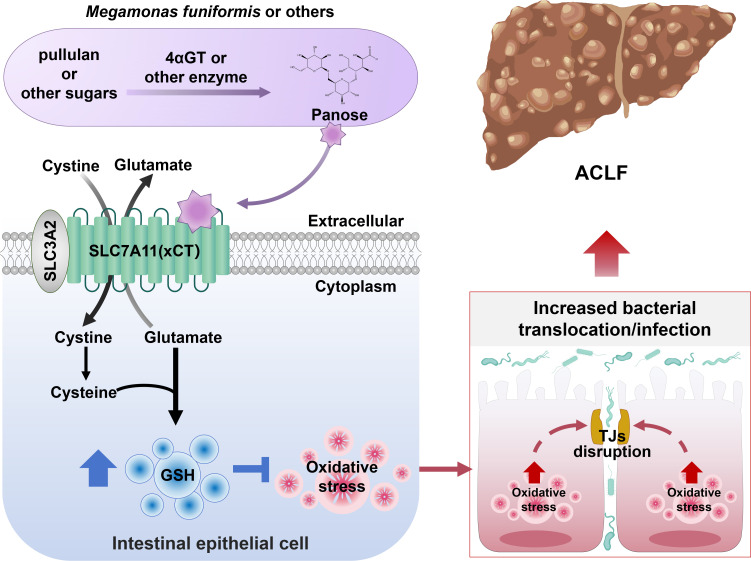

## Introduction

Acute-on-chronic liver failure (ACLF) is often triggered by acute insults in patients with chronic liver disease, leading to rapid liver failure and high mortality (1–3). Among its triggers, bacterial infection is a common and pivotal factor, causing systemic inflammation, hepatocyte death, circulatory dysfunction, and bacterial translocation, which further exacerbate ACLF progression (4–6). A clinical study reported that 37% of patients with ACLF have a bacterial infection at diagnosis, and 46% of the remaining patients develop an infection within 4 weeks (7), highlighting the persistent risk of bacterial infection during ACLF development. Thus, controlling bacterial infection and restoring organ function are crucial for ACLF treatment. Although antibiotics are used to prevent or treat bacterial infection, their overuse and inadequate control of drug-resistant bacteria have considerably increased infections in patients with decompensated cirrhosis. Meanwhile, inappropriate empirical antibiotic regimens can exacerbate clinical outcomes in patients with ACLF (8, 9). Consequently, there is an urgent need for innovative antiinfection strategies to mitigate ACLF progression.

Increased intestinal permeability is a major cause of bacterial infection in patients with cirrhosis, leading to pathogenic bacterial translocation and endotoxemia (10, 11). Various factors lead to intestinal barrier dysfunction, with intestinal oxidative stress being particularly relevant. Oxidative stress causes intestinal barrier impairment by directly oxidizing cell components (proteins, lipids, and DNA) and inducing cell damage (12, 13). Aside from cellular damage, increasing intestinal oxidative stress may decrease the expression of tight junction proteins (14, 15), which are responsible for controlling intestinal permeability (16, 17). Strategies targeting oxidative stress–induced intestinal barrier damage may reduce bacterial translocation and provide promising avenues for mitigating bacterial infection.

The gut microbiota is a potential regulator of ACLF progression, and its metabolites serve as critical mediators of host-microbiota interactions (18–20). Panose, a microbial-derived trisaccharide, consists of 3 glucose units linked by α-1,4 and α-1,6 glycosidic bonds (21). Currently, panose is extensively used in the food industry as an anticariogenic sweetener and an antifading agent. Moreover, panose has prebiotic properties that may selectively promote the growth of beneficial bacteria while inhibiting the proliferation of pathogenic bacteria (22). However, the impact of panose on host biological functions, particularly its potential involvement in regulating ACLF progression, remains to be elucidated.

In this study, we investigated alterations in gut microbial metabolites and found lower fecal panose levels in both infection patients with decompensated cirrhosis and ACLF mice with bacterial infection. Our findings indicated that panose administration mitigated oxidative stress–induced intestinal barrier impairment, reduced pathogenic bacterial translocation, and ameliorated liver injury in ACLF mice. These results highlight the potential of panose in combating bacterial infection and provide a meaningful insight into alleviating ACLF development.

## Results

### Both patients with decompensated cirrhosis and ACLF mice with bacterial infection exhibit lower panose levels.

**Figure 1:**
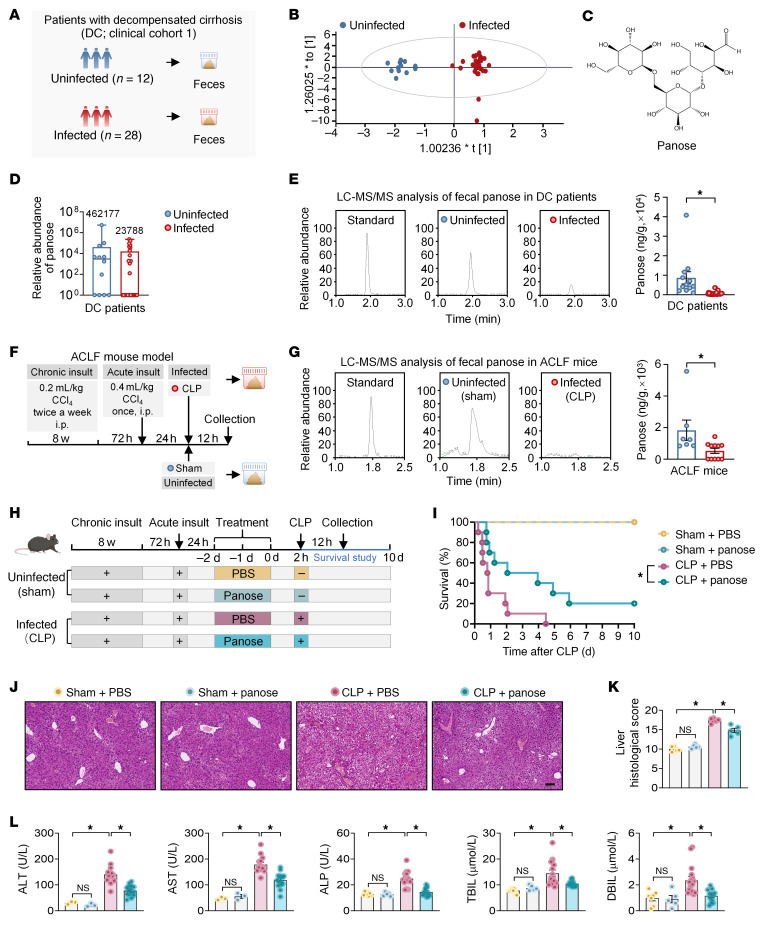
Infected patients with decompensated cirrhosis and ACLF mice exhibit lower panose levels, and panose administration attenuates ACLF progression in mice. (**A**) Schematic diagram of fecal sample collection from infected and uninfected patients with DC. (**B**) Nontargeted metabolite profiles were analyzed using OPLS-DA (*n* = 12–28/group). (**C**) Molecular structure of panose. (**D**) Box plot illustrating the relative abundance of panose in the fecal samples from patients with DC, with mean values indicated on the plot (*n* = 12–28/group). (**E**) Representative chromatograms and fecal panose quantification in patients with DC via LC-MS/MS (*n* = 12–28/group). (**F**) Schematic diagram of the ACLF mouse model. (**G**) Representative chromatograms of fecal panose levels in ACLF mice via LC-MS/MS (*n* = 7–11/group). (**H**) Schematic timeline of panose treatment and survival study in ACLF mice. (**I**) Kaplan-Meier survival curves for ACLF mice treated with PBS or panose (*n* = 5–10/group). (**J**) Representative images of H&E-stained liver sections from ACLF mice. Scale bar: 100 μm. (**K**) Liver histological scores (*n* = 5/group). (**L**) Plasma levels of liver injury markers (ALT, AST, ALP, TBIL, and DBIL) in ACLF mice (*n* = 3–16/group). Data are presented as median ± IQR (**D**) and mean ± SEM (**E**, **G**, **K**, and **L**). Statistical significance was determined by Mann-Whitney *U* test (**E** and **G**), log-rank test (**I**), and 1-way ANOVA with Bonferroni’s post hoc test (**K** and **L**). *P < 0.05. NS, nonsignificant. ACLF, acute-on-chronic liver failure; DC, decompensated cirrhosis; OPLS-DA, orthogonal partial least squares discriminant analysis; LC-MS/MS, liquid chromatography–tandem mass spectrometry; ALT, alanine aminotransferase; AST, aspartate aminotransferase; ALP, alkaline phosphatase; TBIL, total bilirubin; DBIL, direct bilirubin.

Bacterial infection serves as a critical driver of ACLF pathogenesis and also affects gut microbiota and its metabolites (6, 19, 20). To elucidate the gut microbiota metabolic alteration underlying the progression of infection-associated ACLF, we performed nontargeted metabolomics of fecal samples from 12 uninfected and 28 infected patients with decompensated cirrhosis (Figure 1A, further information is provided in Supplemental Table 1, Cohort 1; supplemental material available online with this article; https://doi.org/10.1172/JCI184653DS1). Orthogonal partial least squares discriminant analysis (OPLS-DA) revealed different clustering patterns between groups (Figure 1B), confirming infection-induced remodeling of the fecal metabolome. Notably, panose, a microbial-derived prebiotic, exhibited a declining trend in the feces of infected patients with decompensated cirrhosis (Figure 1, C and D). Quantitative liquid chromatography–tandem mass spectrometry (LC-MS/MS) of the same batch of fecal samples confirmed this finding, indicating that panose levels were markedly decreased in infected patients with decompensated cirrhosis compared with those without infection (Figure 1E).

To confirm the above finding in a murine model, we established an ACLF mouse model following previously reported methods (23), including chronic liver insult, acute liver insult, and bacterial infection induced by cecal ligation and puncture. Control (uninfected) mice underwent chronic liver insult, acute liver insult, and sham surgery (Figure 1F). In line with the human study, ACLF mice exhibited substantially decreased fecal panose levels compared with uninfected counterparts (Figure 1G). These findings suggest that panose may play a potential role in the pathological process of ACLF.

### Panose administration mitigates ACLF in mice.

To investigate the pathophysiological role of panose in the progression of ACLF, panose was administered via gavage to ACLF mice for 3 consecutive days before bacterial infection, followed by an evaluation of their survival time and liver damage (Figure 1H). The survival study showed that panose administration considerably improved survival outcomes in ACLF mice, resulting in a lower mortality rate compared with mice treated with vehicle (PBS) (Figure 1I). Histological examinations revealed extensive hepatic damage in ACLF mice, characterized by swollen, necrotic hepatocytes, along with inflammatory cell infiltration. In contrast, panose treatment effectively alleviated this damage (Figure 1, J and K). Moreover, panose administration substantially reduced plasma levels of liver injury markers, including alanine aminotransferase (ALT), aspartate aminotransferase (AST), alkaline phosphatase (ALP), total bilirubin (TBIL), and direct bilirubin (DBIL) in ACLF mice compared with the PBS-treated mice (Figure 1L).

Given that pulmonary infection is a common infection pathway in clinical patients with ACLF (4), we employed intranasal injection of *Klebsiella pneumoniae* to establish an ACLF mouse model with pulmonary infection and evaluated the potential effect of panose on the liver. As expected, panose administration markedly reduced plasma indicators of liver injury (ALT and AST) and improved pathological hepatic damage compared with PBS treatment (Supplemental Figure 1, A–C). These findings highlight the protective effect of panose in mitigating liver injury in ACLF mice.

### Megamonas funiformis is a potential producer of panose.

**Figure 2:**
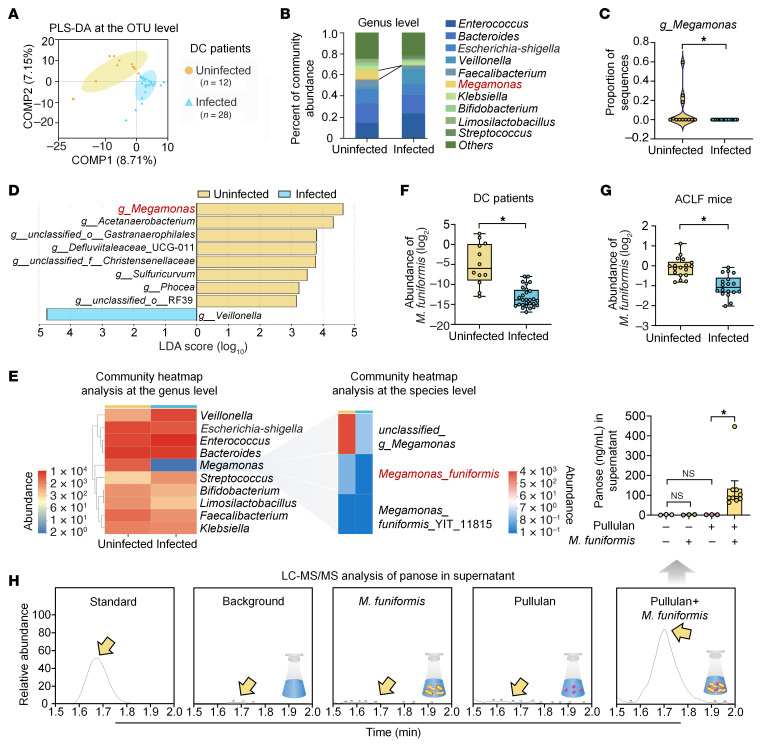
Infected patients with decompensated cirrhosis and ACLF mice have a lower abundance of *M*. *funiformis*, which produces panose from pullulan. (**A**) PLS-DA score plot at the OTU level in fecal samples from patients with DC (*n* = 12–28/group). (**B**) Histogram of microbial community composition at the genus level in fecal samples from patients with DC (*n* = 12–28/group). (**C**) Violin plot illustrating the proportion of *g*_*Megamonas* sequences in fecal samples from patients with DC (*n* = 12–28/group). (**D**) Taxonomic differences at the genus level between groups were analyzed using LEfSe with an LDA score greater than 3 (*n* = 12–28/group). (**E**) Heatmap of the 10 most dominant bacterial genera in fecal samples from patients with DC (left) and *Megamonas* species level (right) (*n* = 12–28/group). (**F**) Relative abundance of *M*. *funiformis* in fecal samples from patients with DC determined by RT-qPCR (*n* = 12–28/group). (**G**) Relative abundance of *M*. *funiformis* in fecal samples from ACLF mice determined by RT-qPCR (*n* = 18/group). (**H**) LC-MS/MS analysis of panose levels in the supernatant of *M*. *funiformis* after 48 hours of culture with pullulan (*n* = 3–9/group). Data are presented as median ± IQR (**C**, **F**, and **G**) and mean ± SEM (**H**). Statistical significance was determined by Mann-Whitney *U* test (**C** and **F**), 2-tailed Student’s *t* test (**G**), and 1-way ANOVA with Fisher’s LSD post hoc test (**H**). *P < 0.05. NS, nonsignificant. DC, decompensated cirrhosis; PLS-DA, partial least squares discriminant analysis; OTU, operational taxonomic unit; LEfSe, linear discriminant analysis effect size; LDA, linear discriminant analysis.

To characterize gut microbiota differences between infected and uninfected patients with decompensated cirrhosis, we performed 16S rRNA gene amplicon sequencing. PLS-DA revealed distinct clustering between the 2 groups (Figure 2A). Despite α-diversity exhibiting no statistical differences (Supplemental Figure 2A), β-diversity displayed considerable variation between the 2 groups (Supplemental Figure 2B). Taxonomic profiling at the genus level identified *Megamonas* as notably depleted in infected patients compared with uninfected controls (Figure 2, B and C). Linear discriminant analysis effect size analysis further confirmed *g*_*Megamonas* as the most discriminative taxon enriched in the uninfected group (Figure 2D). Species-level heatmap analysis specifically highlighted *Megamonas funiformis* (*M*. *funiformis*) as a differentially abundant species, exhibiting reduced fecal abundance in infected patients with decompensated cirrhosis (Figure 2E). RT-qPCR validation consistently showed a lower abundance of *M*. *funiformis* in the feces of both infected patients with decompensated cirrhosis and ACLF mice (Figure 2, F and G).

Given that *Megamonas* spp. participate in carbohydrate fermentation (24, 25), we hypothesized their involvement in panose biosynthesis. To test this hypothesis, we employed a strain of *M*. *funiformis* that exhibited 99.999% average nucleotide identity with the reference genome of *M*. *funiformis* JCM.14723 via whole-genome sequencing (Supplemental Figure 2C). We then cultured *M*. *funiformis* with pullulan, a recognized substrate for panose biosynthesis (26, 27), to assess panose production in vitro. After incubation, LC-MS/MS analysis of the supernatant confirmed panose production from pullulan by *M*. *funiformis* (Figure 2H).

**Figure 3:**
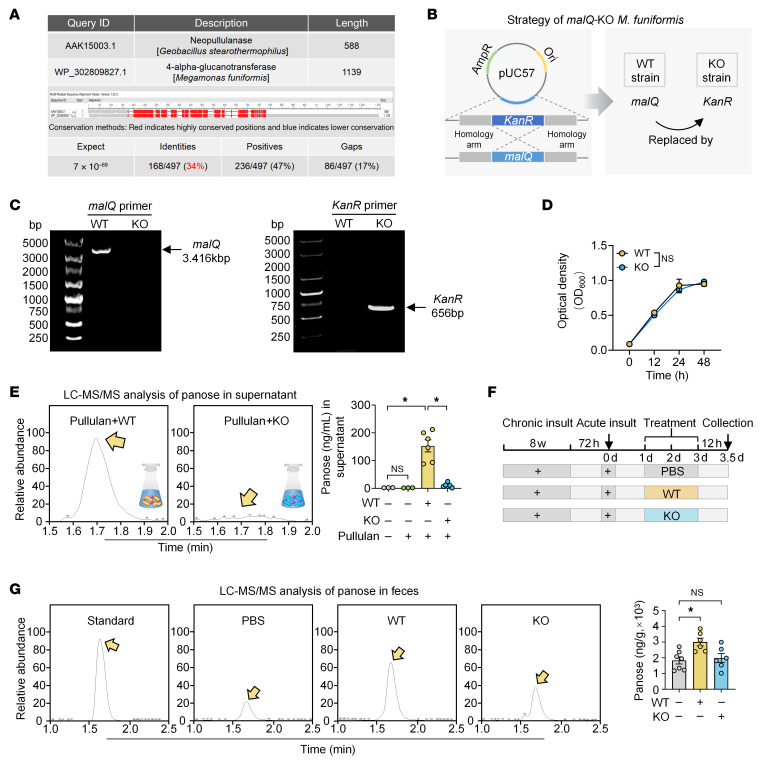
*M*. *funiformis* generates panose from pullulan via 4αGT. (**A**) Protein sequence similarity alignment was assessed by NCBI Protein BLAST. (**B**) The *malQ*-KO strain of *M*. *funiformis* was generated via homologous recombination, replacing the target gene with a *KanR* gene. (**C**) The *malQ*-KO strain of *M*. *funiformis* was confirmed by PCR with *malQ* gene-specific and *KanR* gene-specific primers. (**D**) Growth curves of WT and *malQ*-KO strains (*n* = 3/group). (**E**) LC-MS/MS analysis of panose levels in the supernatant of WT and *malQ*-KO strains after 48 hours of culture with pullulan (*n* = 3–6/group). (**F**) Schematic timeline illustrating the treatment of mice with either WT or *malQ*-KO strain. (**G**) LC-MS/MS analysis of fecal panose levels in mice treated with WT or *malQ*-KO strain (*n* = 6–7/group). Data are presented as mean ± SEM (**D**, **E**, and **G**). Statistical significance was determined by repeated-measures ANOVA (**D**) and 1-way ANOVA with Bonferroni’s post hoc test (**E** and **G**). *P < 0.05. NS, nonsignificant. 4αGT (encoded by *malQ*), 4α-glucanosyltransferase; *KanR*, kanamycin resistance.

To identify the metabolic enzyme responsible for panose biosynthesis in *M*. *funiformis*, we first considered neopullulanase (NPase, EC 3.2.1.135), the most efficient known enzyme for catalyzing the pullulan-to-panose conversion (27). However, NPase has not been identified in *M*. *funiformis*–encoded proteins. Thus, we performed sequence homology screening of *M*. *funiformis*–encoded proteins against NPase. Alignment analysis revealed that 4α-glucanotransferase (4αGT, EC 2.4.1.25, encoded by *malQ*) exhibited the highest sequence homology (34%) to NPase (Figure 3A and Supplemental Figure 2D), implying potential functional overlap.

To validate 4αGT’s role in panose production, we generated a *malQ*-KO *M*. *funiformis* strain and validated its construction via primer-specific amplification (Figure 3, B and C). In vitro experiments demonstrated comparable growth capacity between WT and *malQ*-KO strains (Figure 3D). However, the *malQ*-KO strain exhibited lower efficiency of panose production from pullulan compared with the WT strain (Figure 3E). Consistent with these findings, in vivo supplementation with WT *M*. *funiformis* elevated fecal panose levels in mice, whereas the *malQ*-KO strain did not achieve a statistically significant rise in panose (Figure 3, F and G).

To determine whether the impact of *M*. *funiformis* on panose levels contributes to liver protection, we compared liver injury outcomes in ACLF mice administered WT or *malQ*-KO strains. We found that WT *M*. *funiformis* supplementation markedly attenuated plasma markers of liver injury and improved histopathological scores, whereas the *malQ*-KO strain failed to confer protection (Supplemental Figure 3, A–C). Collectively, these data establish that 4αGT may serve as a pivotal enzyme for panose biosynthesis in *M*. *funiformis*.

### Panose protects against ACLF progression by combating bacterial infection.

We next investigated how panose protects against ACLF. Initial experiments evaluated whether panose alleviates liver damage in the preinfection stage of ACLF, wherein mice experienced chronic and acute liver insult without infection (Supplemental Figure 4A). However, panose treatment failed to alleviate liver injury at this stage, as demonstrated by comparable plasma ALT and AST levels, Sirius Red–stained liver fibrosis scores, and mRNA expression of fibrotic markers (α*-Sma*, *Col1a1*, and *Timp1*) between PBS and panose treatment groups (Supplemental Figure 4, B–D). These observations prompted us to examine whether panose confers protection by modulating bacterial infection.

**Figure 4:**
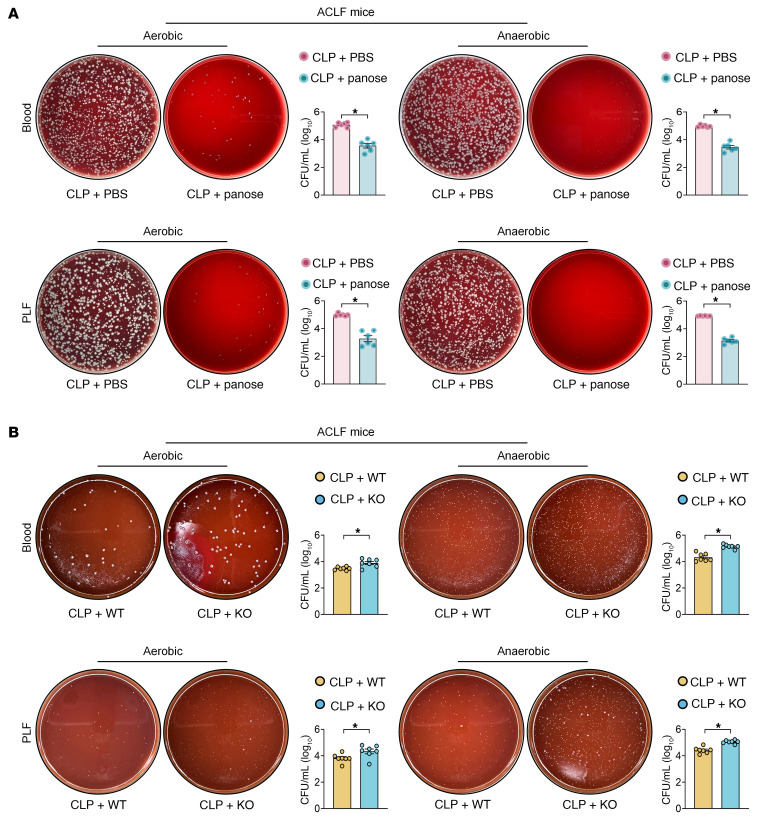
Panose combats bacterial infection in ACLF mice. (**A** and **B**) Representative images and statistical plots of colony formation in peripheral blood and PLF samples after incubation in aerobic or anaerobic conditions. Samples were collected at 12 hours after infection from (**A**) ACLF mice treated with PBS or panose (*n* = 6/group) and (**B**) ACLF mice treated with WT or *malQ*-KO strain (*n* = 7/group). All CFU values were log_10_-transformed. Data are presented as mean ± SEM. Statistical analysis used a 2-tailed Student’s *t* test. *P < 0.05. PLF, peritoneal lavage fluid.

Circulating bacterial load serves as a critical indicator of infection severity. Our data revealed that panose administration substantially reduced bacterial load in peripheral blood and peritoneal lavage fluid (PLF) of ACLF mice compared with PBS-treated controls (Figure 4A). Consistent with this finding, supplementation with the WT strain effectively diminished bacterial load compared with the *malQ*-KO strain in both blood and PLF samples of ACLF mice (Figure 4B). To further validate panose’s antiinfective capacity, we employed 2 established acute infection models: (a) a cecal ligation and puncture–induced polymicrobial infection model and (b) an *E*. *coli*–induced monobacterial infection model. In both acute infection models, panose treatment markedly prolonged survival duration, attenuated liver injury (Supplemental Figure 5, A–H), and reduced systemic bacterial load (Supplemental Figure 6, A and B). Collectively, these findings suggest that panose alleviates ACLF progression, which may stem from its attenuation of bacterial infection.

### Panose combats bacterial infection by restoring the intestinal barrier.

To elucidate how panose reduces bacterial load, we first assessed its direct bactericidal activity. Despite testing concentrations ranging from 50 μM to 1000 μM, panose exhibited no direct antibacterial effects against *E*. *coli* in vitro (Supplemental Figure 7A). Subsequent evaluations of immune cell functions showed that panose neither enhanced phagocytic capacity nor bactericidal activity in neutrophils and macrophages (Supplemental Figure 7B). Moreover, flow cytometry analysis revealed no substantial increase in the percentage of neutrophils or macrophages in panose-treated ACLF mice or mice with acute infection (Supplemental Figure 7, C–H). These negative results directed our focus toward panose’s potential role in regulating intestinal barrier function to limit bacterial translocation.

**Figure 5:**
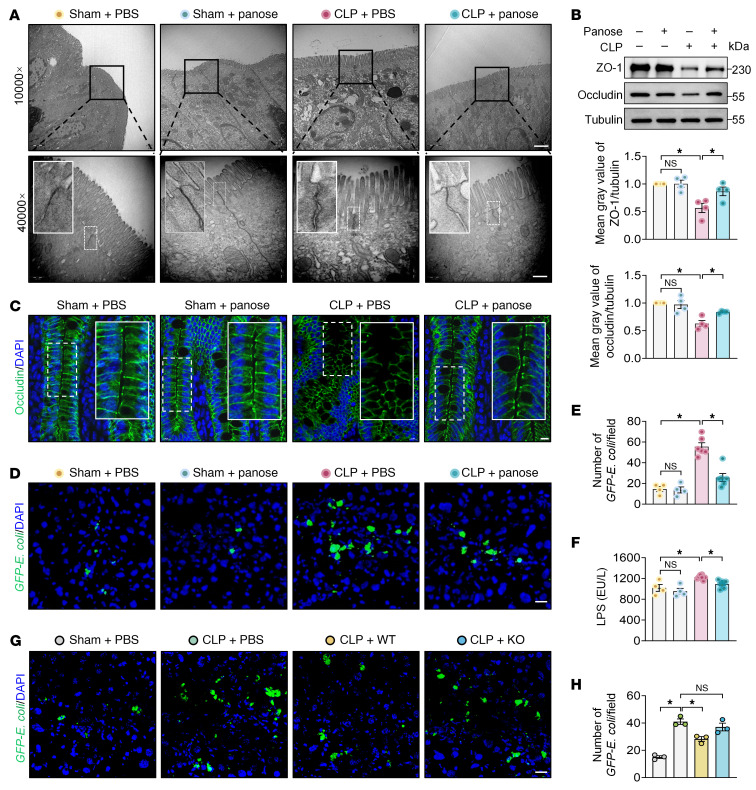
Panose combats bacterial infection in ACLF mice by restoring the intestinal barrier. (**A**) Representative TEM images of ileum TJs in ACLF mice. Scale bars: 2 μm (10,000× original magnification) and 500 nm (40,000× original magnification). (**B**) Western blot analysis and quantification of ZO-1 and occludin protein expression in the ileum of ACLF mice (*n* = 4/group). (**C**) Representative immunofluorescence images of occludin in the ileum of ACLF mice (green: occludin; blue: DAPI-stained nuclei). Scale bar: 10 μm. (**D** and **G**) Representative images of *GFP–E*. *coli* fluorescence intensity in the liver of ACLF mice (green: *GFP–E*. *coli*; blue: DAPI-stained nuclei). Scale bar: 20 μm. (**E**) Quantification of *GFP–E*. *coli* fluorescence signals in the liver tissue from PBS- or panose-treated ACLF mice (*n* = 4–6/group). (**F**) LPS levels in the plasma of ACLF mice at 12 hours after infection (*n* = 4–8/group). (**H**) Quantification of *GFP–E*. *coli* fluorescence signals in the liver tissue from ACLF mice treated with WT or *malQ*-KO strain (*n* = 3/group). Data are presented as mean ± SEM. Statistical significance was determined by 1-way ANOVA with Bonferroni’s post hoc test. *P < 0.05. NS, nonsignificant. TEM, transmission electron microscopy; TJs, tight junctions; ZO-1, zonula occludens-1.

The intestinal barrier’s structural integrity critically depends on the tight junction structure, which governs paracellular permeability by sealing intercellular spaces between epithelial cells (28, 29). Transmission electron microscopy analysis revealed severe tight junction structure disruption in ACLF mice, characterized by blurred junctional architecture and abnormally widened intercellular gaps. However, panose treatment substantially ameliorated these structural defects (Figure 5A). Zonula occludens-1 (ZO-1) and occludin are critical tight junction–associated proteins essential for maintaining intestinal homeostasis (30). Western blotting revealed the restoration of ZO-1 and occludin expression in intestinal tissues from panose-treated ACLF mice compared with PBS controls (Figure 5B). Likewise, immunofluorescence staining further confirmed that panose preserved occludin localization at the tight junction structure more effectively than PBS treatment (Figure 5C).

To evaluate the functional consequences of tight junction structure restoration, we tracked *GFP–E*. *coli* translocation to the liver in ACLF mice. Panose treatment considerably reduced hepatic fluorescence intensity compared with PBS controls in ACLF mice with abdominal or pulmonary bacterial infection (Figure 5, D and E, and Supplemental Figure 1D). Meanwhile, plasma LPS levels verified that panose effectively reduced the leakage of intestinal bacterial toxins into the circulation (Figure 5F). Similarly, administration of WT *M*. *funiformis*, but not the *malQ*-KO strain, diminished *GFP–E*. *coli* fluorescence signals in the livers of ACLF mice (Figure 5, G and H). In addition, panose also maintained tight junction integrity and reduced bacterial translocation in acute polymicrobial-infected mice (Supplemental Figure 8, A–F). These data collectively indicate that panose’s antiinfective effects correlate with intestinal barrier restoration.

### Panose ameliorates gut barrier dysfunction by inhibiting oxidative stress.

**Figure 6:**
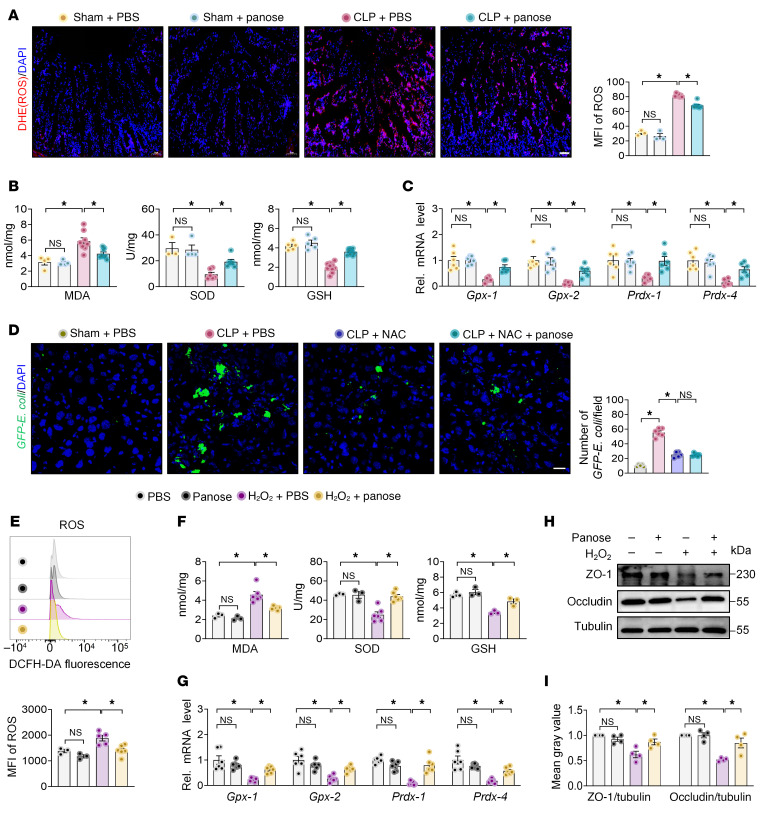
Panose ameliorates gut barrier dysfunction by inhibiting oxidative stress. (**A**) ROS levels in ileum sections of ACLF mice were visualized with DHE staining and quantified using MFI (red: ROS; blue: DAPI-stained nuclei). Scale bar: 50 μm (*n* = 3–7/group). (**B**) MDA, SOD, and GSH levels in the ileum tissue of ACLF mice (*n* = 3–10/group). (**C**) The mRNA levels of antioxidant markers (*Gpx-1*, *Gpx-2*, *Prdx-1*, and *Prdx-4*) in the ileum tissue were determined by RT-qPCR (*n* = 6/group). (**D**) Representative images and quantification of *GFP–E*. *coli* fluorescence intensity in the liver of ACLF mice after ROS elimination via NAC (green: *GFP–E*. *coli*; blue: DAPI-stained nuclei). Scale bar: 20 μm (*n* = 5–6/group). (**E**–**I**) Mode-K cells were exposed to H_2_O_2_-induced oxidative stress for 12 hours before sample collection and analysis. (**E**) Intracellular ROS levels were assessed using DCFH-DA staining, measured by flow cytometry, and quantified with MFI (*n* = 3–6/group). (**F**) MDA, SOD, and GSH levels in the Mode-K cells (*n* = 3–6/group). (**G**) The mRNA levels of antioxidant markers (*Gpx-1*, *Gpx-2*, *Prdx-1*, and *Prdx-4*) in the Mode-K cells were determined by RT-qPCR (*n* = 6/group). (**H**) Western blot analysis and (**I**) quantification of ZO-1 and occludin protein expression (*n* = 4/group). Data are presented as mean ± SEM. Statistical significance was determined by 1-way ANOVA with Bonferroni’s post hoc test. *P < 0.05. NS, nonsignificant. DHE, dihydroethidium; MDA, malondialdehyde; SOD, superoxide dismutase; GSH, glutathione; *Gpx-1,* glutathione peroxidase 1; *Gpx-2,* glutathione peroxidase 2; *Prdx-1*, peroxiredoxin 1; *Prdx-4,* peroxiredoxin 4; NAC, *N*-acetylcysteine; DCFH-DA, 2′,7′-dichlorodihydrofluorescein diacetate.

Epithelial cell apoptosis, inflammation, and oxidative stress constitute key drivers of intestinal barrier disruption (31–35). However, TUNEL assays detected no statistical differences in intestinal apoptotic cell counts between the panose and PBS treatment groups in either ACLF or acute polymicrobial-infected mice (Supplemental Figure 9, A and B). Similarly, panose treatment did not affect intestinal lamina propria inflammation levels in both ACLF mice and acute polymicrobial-infected mice compared with PBS treatment (Supplemental Figure 9, C and D). To assess whether panose attenuates intestinal barrier disruption by reducing oxidative stress, we measured intestinal ROS levels using the dihydroethidium (DHE) probe. We observed that panose substantially reduced ROS levels in the small intestine villi of ACLF mice compared with PBS controls (Figure 6A). Simultaneously, panose reduced malondialdehyde (MDA) levels while elevating antioxidant levels such as superoxide dismutase (SOD) and glutathione (GSH) in intestinal tissue (Figure 6B). In addition, panose reversed the downregulation of antioxidant genes (*Gpx-1*, *Gpx-2*, *Prdx-1*, and *Prdx-4*) in the intestines of ACLF mice (Figure 6C). Likewise, these results were validated in the acute polymicrobial-infected mice (Supplemental Figure 10, A–C).

To further elucidate the impact of oxidative stress on panose-mediated restoration of the intestinal barrier, we used *N*-acetylcysteine (NAC) to inhibit oxidative stress in the intestine. The NAC administration notably reduced *GFP–E*. *coli* translocation to the liver in ACLF mice. However, coadministration of panose with NAC provided no additive benefits in reducing bacterial translocation in ACLF mice and acute polymicrobial-infected mice (Figure 6D and Supplemental Figure 10D). These data indicate that panose restores the intestinal barrier at least in part through its antioxidative effects.

Then, we employed the Mode-K cell line from the small intestine to validate the above findings. The Cell Counting Kit-8 assay indicated that 50 μM panose was safe for Mode-K cells (Supplemental Figure 11A). Upon exposure to H_2_O_2_-induced oxidative stress, we observed notable elevations in oxidative indicators (such as ROS and MDA) alongside compromised antioxidant defense systems (including GSH, SOD, and antioxidant genes) in Mode-K cells. However, treatment with panose effectively reversed the above phenomena (Figure 6, E–G). Moreover, panose reversed the H_2_O_2_-induced reduction in ZO-1 and occludin protein expression in Mode-K cells (Figure 6, H and I). Together, these findings indicate that panose ameliorates the dysregulation of tight junction structure by inhibiting oxidative stress.

### Panose reduces intestinal oxidative stress by interacting with the xCT protein.

**Figure 7:**
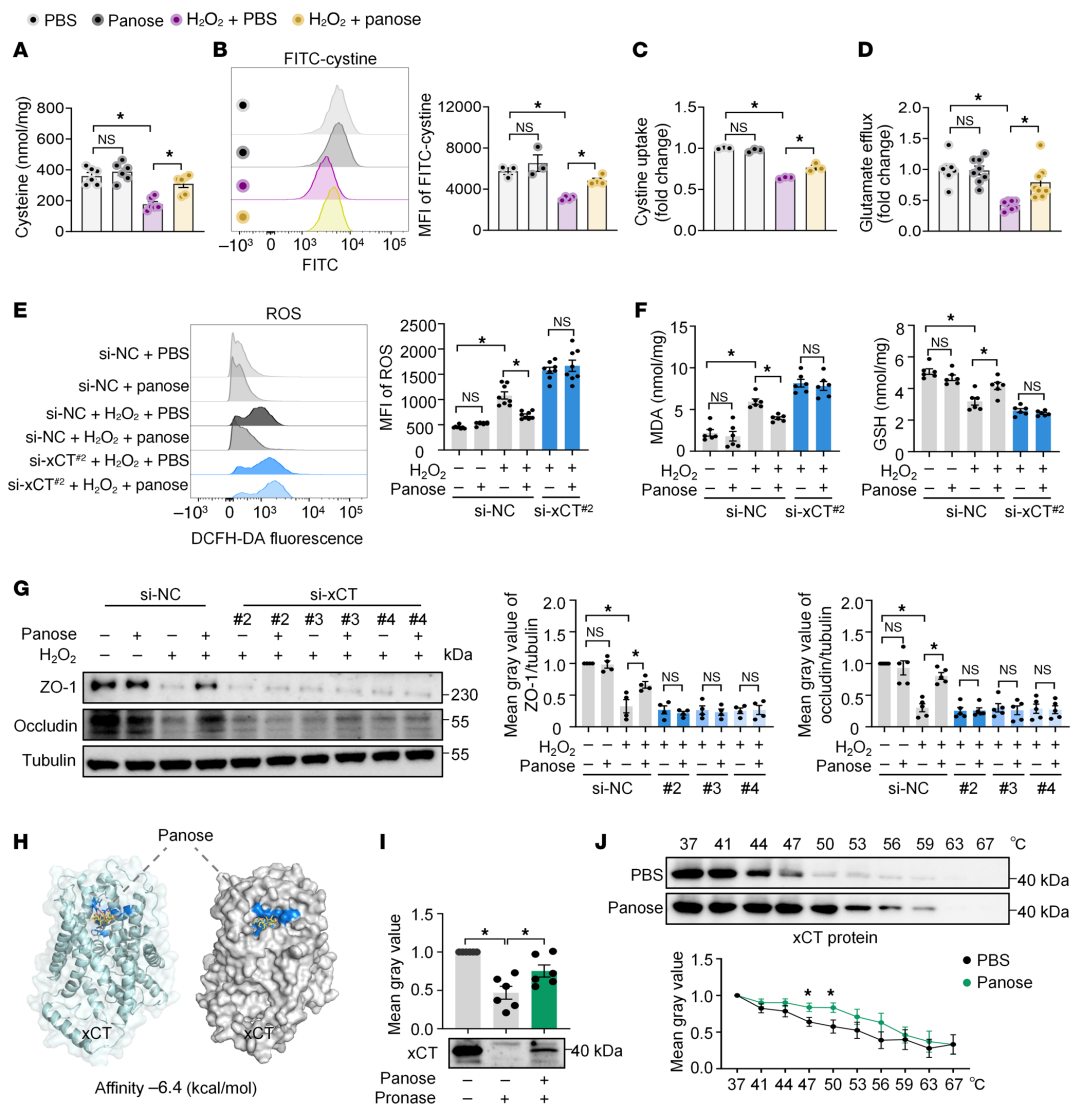
Panose reduces intestinal oxidative stress by interacting with the xCT protein. (**A**–**G**) Mode-K cells were exposed to H_2_O_2_-induced oxidative stress for 12 hours before sample collection and analysis. (**A**) Intracellular cysteine levels (*n* = 6/group). (**B**) FITC-cystine uptake was measured by flow cytometry and quantified using MFI (*n* = 3–4/group). (**C**) Cystine uptake was analyzed using a fluorescent enzyme labeler at 490/535 nm (*n* = 3/group). (**D**) Glutamate efflux levels in the media (*n* = 8/group). (**E**–**G**) Mode-K cells transfected with si-NC or si-xCT were analyzed as follows: (**E**) Intracellular ROS levels were assessed using DCFH-DA staining, measured by flow cytometry, and quantified with MFI (*n* = 8/group). (**F**) MDA and GSH levels (*n* = 6/group). (**G**) Western blot analysis and quantification of ZO-1 and occludin protein expression (*n* = 4–5/group). (**H**) Schematic depiction of molecular docking between panose and the extracellular structural domain of the xCT protein (PDB: 7EPZ, B chain), with a docking affinity of –6.4 kcal/mol. (**I**) The DARTS experiment was conducted to assess the panose-xCT protein interaction (*n* = 6/group). (**J**) The CETSA experiment was conducted to assess the panose-xCT protein interaction (*n* = 9/group). Data are presented as mean ± SEM. Statistical significance was determined by 1-way ANOVA with Bonferroni’s post hoc test (**A**–**G**, and **I**) and 2-tailed Student’s *t* test (**J**). *P < 0.05. NS, nonsignificant. xCT (known as SLC7A11), solute carrier family 7 member 11; si-NC, negative control siRNA; si-xCT, xCT-specific siRNA; DARTS, drug affinity responsive target stability; CETSA, cellular thermal shift assays.

GSH is a pivotal antioxidant essential for oxidative stress neutralization, and cysteine is a key limiting substrate for GSH synthesis (36, 37). We observed that panose markedly reversed H_2_O_2_-induced cysteine depletion in Mode-K cells (Figure 7A), suggesting its regulatory role in maintaining GSH synthesis. The cellular availability of cysteine is critically regulated by the Xc^–^ system, a cystine/glutamate antiporter that imports extracellular cystine in exchange for intracellular glutamate, thereby enabling rapid conversion of cystine to cysteine for GSH production (38). To investigate whether panose affects the activity of the Xc^–^ system, we measured cystine uptake and glutamate efflux capacity in Mode-K cells. Our data revealed that H_2_O_2_ stimulation considerably impaired cystine uptake, whereas panose treatment reversed this effect (Figure 7, B and C). Meanwhile, panose increased the glutamate efflux capacity in the H_2_O_2_-stimulated Mode-K cells (Figure 7D). These findings suggest that panose activates the Xc^–^ system, thereby increasing cysteine availability and subsequent GSH biosynthesis.

The Xc^–^ system is composed of 2 subunits: the light-chain solute carrier family 7 member 11 (SLC7A11, also known as xCT) and the heavy-chain solute carrier family 3 member 2 (SLC3A2, also known as 4F2hc). xCT mediates cystine uptake in exchange for intracellular glutamate, a critical mechanism for cellular antioxidant defense. 4F2hc acts as a chaperone that stabilizes xCT and regulates its membrane trafficking (39, 40). Hence, we hypothesized that xCT serves as the primary target protein mediating the protective effects of panose against intestinal oxidative stress. To validate this hypothesis, we designed 4 siRNAs targeting xCT (si-xCT). Immunoblot analysis confirmed that 3 siRNAs effectively suppressed xCT expression in Mode-K cells (Supplemental Figure 11B). In the group transfected with negative control (si-NC), panose treatment substantially alleviated oxidative stress in H_2_O_2_-stimulated Mode-K cells, as evidenced by decreased ROS and MDA levels, alongside increased GSH levels, Xc^−^ system activity, and intracellular cysteine levels. However, these beneficial effects of panose were abolished in si-xCT–transfected Mode-K cells (Figure 7, E and F, and Supplemental Figure 11, C–E). In addition, panose rescued H_2_O_2_-induced downregulation of tight junction proteins in si-NC–transfected cells but not in si-xCT–transfected cells (Figure 7G).

To validate these findings, we overexpressed xCT using a plasmid (ov-xCT), with transfection efficiency confirmed by immunoblotting (Supplemental Figure 12A). As expected, panose further improved Xc^–^ system activity, elevated cysteine levels, and reduced oxidative stress levels in ov-xCT–transfected cells compared with PBS-treated controls during H_2_O_2_ stimulation (Supplemental Figure 12, B–G). Meanwhile, panose treatment showed a tendency to further increase the expression of ZO-1 and occludin in H_2_O_2_-stimulated ov-xCT–transfected cells relative to the PBS-treated controls (Supplemental Figure 12H). Given that panose does not affect the protein expression of xCT and 4F2hc (Supplemental Figure 13A), our results reinforce the concept that panose mitigates oxidative stress–induced intestinal barrier injury primarily through modulating xCT protein activity.

To confirm whether panose directly interacts with xCT to modulate its enzymatic activity, we performed molecular docking analysis. As illustrated in Figure 7H, panose exhibited binding affinity to the extracellular domain of xCT, achieving an affinity of –6.4 kcal/mol. To validate this interaction, we employed 2 biophysical assays: drug affinity responsive target stability (DARTS) (41) and cellular thermal shift assay (CETSA) (42). DARTS analysis demonstrated that panose markedly attenuated pronase-mediated hydrolysis of xCT compared with the control (Figure 7I). Likewise, CETSA revealed enhanced thermal stability of xCT in panose-treated Mode-K cells (Figure 7J). These results confirmed direct binding between panose and xCT. In contrast, panose showed no effects on 4F2hc proteolysis or thermostability (Supplemental Figure 13, B and C). Collectively, these findings demonstrate that panose mitigates intestinal oxidative stress through interaction with xCT.

### The translational potential of panose in the context of ACLF.

Finally, we investigated the translational potential of panose. Distinct from conventional antibiotics that directly target pathogens, panose combats bacterial infection through intestinal barrier restoration. This mechanistic divergence prompted us to investigate the potential synergism between panose and antibiotic (e.g., norfloxacin) (43). We tested 2 combination strategies: sequential administration and preinfection combined administration. Our observations indicated that, compared with the PBS treatment group, both combined treatment strategies markedly extended survival time and reduced liver damage in ACLF mice. In comparison to monotherapy groups, these combined strategies also demonstrated trends toward enhanced survival and reduced liver damage, although these differences did not reach statistical significance (Supplemental Figure 14, A–D). These findings suggest that the combination of panose and antibiotic may exhibit synergistic potential in alleviating ACLF progression.

To investigate the association between fecal panose levels and ascites volume, we enrolled 25 additional uninfected patients with decompensated cirrhosis, including 12 with ascites and 13 without ascites (Supplemental Figure 15A; further information is provided in Supplemental Table 2, Cohort 2). LC-MS/MS quantification demonstrated lower fecal panose levels in patients with ascites compared with patients without ascites (Supplemental Figure 15B). Based on this finding, we attempted to detect whether panose could improve ascites in a murine model. Since the current ACLF mouse model did not show apparent ascites accumulation, we conducted an advanced liver disease model with minor modifications to the method described by Nautiyal et al. (44). This optimized model successfully induced visible ascites (Supplemental Figure 15, C–E), but panose treatment failed to improve ascites volume and liver injury (Supplemental Figure 15, F–J), indicating the limitation of panose as a monotherapy for advanced liver disease with ascites.

Given the prohibitively complex purification process and substantial production costs associated with panose, clinical translation using purified panose remains challenging. Therefore, we explored isomalto-oligosaccharide (IMO), a commercially viable panose-containing compound offering greater practical feasibility (45). Results demonstrated that IMO supplementation prolonged survival time in ACLF mice compared with untreated controls (Supplemental Figure 16, A and B). Similarly, in acute polymicrobial-infected mice, IMO administration conferred dose-dependent survival benefits relative to the PBS-treated group (Supplemental Figure 17A). Furthermore, IMO treatment suppressed bacterial translocation to the liver, decreased plasma LPS levels, reduced plasma levels of ALT, and mitigated circulating bacterial load in ACLF mice (Supplemental Figure 16, C–G). Parallel experiments in the acute polymicrobial-infected mice corroborated IMO’s capacity to mitigate hepatic injury and intestinal bacterial translocation in the liver (Supplemental Figure 17, B and C). In addition, analysis of the key active components of IMO, which include panose, isomaltotriose, isomaltotetraose, and isomaltose, confirmed that panose played a crucial role in IMO’s protective actions (Supplemental Figure 17 D), suggesting the potential feasibility of utilizing IMO as an alternative reagent to panose for further research.

## Discussion

ACLF is predominantly precipitated by bacterial infection, emphasizing the critical need to promptly and effectively prevent bacterial infection (4). In this study, we observed lower fecal panose levels in both infection patients with decompensated cirrhosis and ACLF mice with bacterial infection compared with uninfected controls. Further animal studies demonstrated that panose administration substantially alleviated liver injury and extended survival in ACLF mice by decreasing bacterial infection. Mechanistically, panose interacted with xCT in small intestinal epithelial cells, promoting GSH production to mitigate oxidative stress–induced gut barrier damage. Our findings highlight the potential of panose in combating bacterial infection and alleviating ACLF progression.

NPase has been identified as a highly efficient enzyme for catalyzing the pullulan-to-panose conversion (27), and it has been predominantly observed in *Bacillus pseudofirmus* 703 (46), *Bacillus* sp. KSM-1876 (47), and *Rhodothermus marinus* (48). In this study, we discovered the 4αGT enzyme in *M*. *funiformis* that shares 34% sequence similarity with NPase and demonstrates catalytic activity in converting pullulan to panose. However, the in vitro conversion efficiency of pullulan to panose by *M*. *funiformis* appeared suboptimal under current experimental conditions. Three potential factors may explain this limitation: (a) the relatively short incubation period (48 hours) might not allow complete substrate conversion; (b) the bacterial biomass may have been insufficient relative to the substrate concentration employed; (c) the enzymatic activity of 4αGT was possibly low within the current culture system. Furthermore, aside from pullulan, we have not examined whether *M*. *funiformis* may employ alternative substrates to synthesize panose, which warrants future investigation. Given the functional redundancy in carbohydrate metabolism exhibited by the gut microbiota, *M*. *funiformis* is probably only one member of a diverse microbial community that collectively influences intestinal panose levels. Future research will likely reveal additional panose-producing microbiota, thus further elucidating the mechanisms behind changes in panose levels.

Oxidative stress is one of the central drivers of intestinal barrier compromise (13). Our study suggests that panose mitigates ROS-induced barrier damage by directly binding to xCT and enhancing its activity, resulting in higher antioxidant GSH levels. Although the extracellular localization of the panose-xCT interaction was established, the mechanism by which this hydrophilic molecule augments the enzymatic activity of xCT’s intracellular N-terminal domain remains unclear. Previous studies indicated that phosphorylation regulates xCT N-terminal enzymatic activity (49). Thus, we speculate that the binding of panose to xCT’s extracellular region may trigger a conformational shift in the protein, affecting the level of posttranslational modifications and consequently activating its N-terminus. Further structural and biochemical studies are required to fully understand how panose regulates xCT enzymatic activity.

We noted that panose effectively reduced bacterial infection through a mechanism distinct from antibiotics, repairing the intestinal barrier rather than sterilizing pathogens. Despite our preliminary findings indicating that the combination of panose with norfloxacin showed a tendency for extended survival and decreased hepatic damage in comparison to the monotherapy groups, the observed trends did not achieve statistical significance. This limitation highlights the need for further studies to confirm these findings, particularly by employing larger sample sizes to increase statistical power, experimenting with varied dosing regimens to optimize therapeutic effects, and exploring combinations with other antibiotics to identify synergistic interactions.

Despite our clinical observation of reduced fecal panose levels in patients with decompensated cirrhosis and ascites, panose treatment failed to improve ascites volume and liver injury in the murine ascites model. This discrepancy may arise from several factors. First, the multifactorial pathogenesis of ascites (involving portal hypertension, systemic inflammation, vascular permeability, and liver dysfunction, etc.) may not be fully addressed by barrier-focused interventions. Second, panose depletion may be a downstream consequence of microbial imbalance rather than having a direct causal link to ascites. Third, the murine model may not adequately capture the complexity of human disease, and species-specific differences could influence the efficacy of pannose. Last, the human sample size was limited, requiring a larger cohort to validate the correlation between panose and ascites volume. Our data revealed the limitation of panose as a monotherapy for advanced liver disease with ascites.

Although panose is a safe food ingredient, high production costs limit its application in the clinic. Consequently, we investigated IMO, a cost-effective and panose-containing prebiotic. IMO has been thoroughly investigated for its various biological effects, which include promoting the growth of beneficial gut bacteria, alleviating constipation, improving hyperlipidemia, and enhancing immune responses (50–53). Our investigation demonstrated that IMO exhibited similar effects to panose in reducing pathological bacterial translocation, ameliorating liver injury, and prolonging survival time in ACLF mice. Importantly, analysis of the key active components of IMO showed that panose played a crucial role in driving IMO’s protective effects. These findings suggest that IMO may serve as a viable alternative to panose for further research, particularly in contexts where panose availability or cost may be limiting factors. In addition, we observed that isomaltotriose, a trisaccharide component similar in structure to panose, also exhibited beneficial effects in improving survival rates in acute polymicrobial-infected mice (Supplemental Figure 17E). This observation underscores the potential protective value of trisaccharide-based small molecules in combating bacterial infection.

In conclusion, our findings indicate that panose possesses unique potential as a gut barrier–protective agent in preventing infections, providing a potential strategy for ACLF prevention.

## Methods

Detailed methods can be found in Supplemental Methods.

### Sex as a biological variable.

The human study included participants of both sexes. The mouse model used only males to minimize variability and ensure consistent model behavior, as male individuals typically exhibit more severe infections and clinical features relevant to the disease, and females are more resistant to infections (54, 55).

### Patient characteristics.

Patients hospitalized with acute decompensated cirrhosis were included in the study. Cohort 1 consisted of 40 patients with decompensated cirrhosis, including 12 without bacterial infection and 28 with bacterial infection. Cohort 2 consisted of 25 patients with decompensated cirrhosis without bacterial infection, categorized as 13 without ascites and 12 with ascites. Exclusion criteria were the following: (a) age younger than 18 years or older than 80 years; (b) malignancy; (c) severe chronic extrahepatic disease with a poor short-term prognosis; (d) receipt of immunosuppressive agents for nonhepatic diseases; (e) HIV infection. Additional patient information is available in Supplemental Tables 1 and 2.

### Animal studies.

Specific pathogen–free male C57BL/6J mice, aged 6–8 weeks, were obtained from SiPeiFu Laboratory Animal Technology Co. Before the experiment, all mice were acclimatized to a controlled environment (12-hour light/12-hour dark cycle) for 1 week and had free access to food and water. Further details on the mouse model and the methods of pharmacological intervention can be found in the Supplemental Methods.

### Bacterial experiment.

*M*. *funiformis* (JCM, 14723; GDMCC, 1.1303) was obtained from the Microbial Culture Collection Center and cultured under strict anaerobic conditions (10% H_2_, 10% CO_2_, and 80% N_2_) at 37°C in brain-heart infusion broth (Huankai, 024053). Additional bacterial experiments can be found in the Supplemental Methods.

### Cell culture.

The Mode-K cell line from intestinal epithelial (BeNa Culture Collection, BNCC338300) was cultured in DMEM (Gibco, C11995500BT) with 10% FBS (Gibco, 10099141C) and 1% penicillin and streptomycin (Gibco, 15140122) at 37°C in a humidified atmosphere with 5% CO_2_. The culture medium was changed daily and passaged every 2 days. Further experimental details can be found in the Supplemental Methods. The interference sequences targeting xCT are listed in Supplemental Table 5.

### Immunoblot analysis.

Protein lysates were prepared using RIPA buffer (Beyotime, P0013B) with phosphatase and protease inhibitors (Beyotime, P1046). Proteins were separated by SDS-PAGE and transferred to PVDF membranes (MilliporeSigma). Membranes were blocked with 5% nonfat milk (Solarbio, D8340) at room temperature for 1 hour, then incubated with primary antibodies overnight at 4°C. After washing with 1× TBST (Biosharp, BL346A), HRP-conjugated secondary antibodies were applied at room temperature for 1 hour. Signals were visualized using a ChemiDoc MP system (Bio-Rad) and quantified with ImageJ version 1.53 (NIH). Antibody details are provided in Supplemental Table 6.

### RT-qPCR.

RNA samples were extracted from tissue and cells using RNAiso Plus (TaKaRa, 9109) and reverse-transcribed into cDNA with the ReverTra Ace qPCR RT Kit (TOYOBO, FSQ-101). The cDNA was used for RT-qPCR on an ABI 7500 system (Applied Biosystems) with SYBR Green Realtime PCR Master Mix Plus (TOYOBO, QPK-201) and specific primers (listed in Supplemental Table 7). Housekeeping controls were 18S rRNA and 16S rRNA. Relative mRNA expression levels were determined using the 2^–ΔΔCt^ method.

### Statistics.

Statistical analysis was conducted using GraphPad Prism 10. Data values are expressed as mean ± SEM unless stated otherwise. For 2-group comparisons, 2-tailed Student’s *t* test was employed for normally distributed data; the Mann-Whitney *U* test was used for nonnormally distributed data. Unless stated otherwise, 1-way ANOVA with Bonferroni’s post hoc test was applied for comparisons involving more than 2 groups, and the log-rank test was applied for survival rates. *P* values less than 0.05 were considered statistically significant for all analyses, and sample sizes are provided in the figure legends.

### Study approval.

All human studies were approved by the Ethical Committee of Nanfang Hospital, Southern Medical University (NFEC-2020-255 and NFEC-2019-035). Informed consent was obtained from each participant before enrollment.

All animal experiments received approval from the IACUC of Southern Medical University (approval SMUL2022309 and SMUL202310024).

### Data availability.

16S rRNA gene sequencing data have been deposited into the NCBI Sequence Read Archive repository under BioProject number PRJNA1094620. Complete genome sequencing data of *M*. *funiformis* have been submitted to NCBI under BioProject number PRJNA1208281. Data values for all graphs in the manuscript and supplemental materials are reported in the Supporting Data Values file. Unedited gel images are available in the supplement, and additional reanalysis information can be obtained from the lead contact, Peng Chen.

## Author contributions

JL, SX, MC, YC, and FL performed the experiments and analyzed the data. CH collected human samples and analyzed clinical data. NT, TC, LZ, and JB supported the animal experiments and project management. WZ and HP participated in the bacterial culture and analysis. PG participated in the bacterial gene editing experiment. JL and PC interpreted the data and drafted and edited the manuscript. BS, JC, and PC designed and supervised the study. All authors reviewed the manuscript.

## Supplementary Material

Supplemental data

Unedited blot and gel images

Supporting data values

## Version 1 06/06/2025

In-Press Preview

## Version 2 07/15/2025

Electronic publication
