# Supplementary material for: Panose prevents acute-on-chronic liver failure by reducing bacterial infection in mice
Source: J Clin Invest. 2025 Jun 3;135(14):e184653. doi: 10.1172/JCI184653 (PMC12259250; doi:10.1172/JCI184653)
Supplement: Supplemental data [file jci-135-184653-s255.pdf]

Supplementary Methods

1.Non-targeted metabolomics

Fecal samples were collected from patients with decompensated cirrhosis (DC). Each lyophilized sample (20 mg) was homogenized in 900 μL of 80% methanol containing 0.1% formic acid. After vortex mixing, samples were precipitated at -20 °C overnight, followed by centrifugation (12,000 g, 15 min, 4 °C). The supernatant underwent secondary precipitation (4 °C, 12 h) and centrifugation under identical conditions. Quality control (QC) was generated by combining 20 μL aliquots from each specimen.

Metabolite separation was achieved using a Waters ACQUITY UPLC I-Class system with a Synapt G2 HDMS mass spectrometer and equipped with a HSS T3 C18 column (2.1 × 100 mm, 1.8 μm) maintained at 40 °C. The mobile phase (0.4 mL/min) consisted of 0.1% formic acid in water (A) and acetonitrile (B). Mass spectrometry was performed in dual ESI mode with the following parameters: capillary voltages at 3.0 kV (ESI+)/2.5 kV (ESI−), cone voltage at 30 V, collision energy of 20-35 eV, desolvation gas 1,000 L/h at 550 °C, cone gas flow rate at 150 L/h, and source temperature of 120 °C.

Raw data processing via Progenesis QI (version 2.4, Waters, USA) included feature detection, retention time alignment, and compound annotation using HMDB databases. Orthogonal partial least squares discriminant analysis (OPLS-DA) was performed using SIMCA-P software (version 14.1, Umetrics, Sweden).

2.Targeted quantification of panose

Fecal samples were homogenized in ultrapure water (1:9, w/v) and aliquots (300 μL) were mixed with 90% acetonitrile (1:4, v/v). After 2 h of incubation at -80 °C, samples were centrifuged (16,000 g, 10 min, 4 °C). The supernatant underwent overnight incubation at -80 °C followed by centrifugation under identical conditions. The resulting supernatant was nitrogen-evaporated and reconstituted in 100 or 150 μL of 50% acetonitrile prior to 0.22 μm membrane filtration. Bacterial culture supernatants were precipitated with 90% acetonitrile (1:4, v/v) at -80 °C for 2 h, followed by centrifugation (16,000 g, 10 min, 4 °C).

The supernatant was incubated overnight at -80 °C and recentrifuged under identical conditions. The resulting supernatant was lyophilized and redissolved in 50% acetonitrile (volume adjusted to ensure complete dissolution) with vortex-assisted mixing. After centrifugation, the solution was filtered through a 0.22 μm membrane, and 100 μL aliquots were analyzed.

Panose quantification was performed using liquid chromatography-mass spectrometry (LC-MS/MS). Processed sample extracts were analyzed on a Thermo Scientific Prelude SPLC system coupled to a TSQ Vantage triple quadrupole mass spectrometer. Chromatographic separation was achieved using an Amide column (1.7 μm, 2.1 × 100 mm, Waters, USA) maintained at 40 °C with isocratic elution (0.3 mL/min) using an acetonitrile/water mobile phase. Mass spectrometric detection employed electrospray ionization with alternating spray voltages (3.0 kV positive/2.5 kV negative). Operational parameters were optimized as follows: sheath gas 45 Arb, auxiliary gas 8 Arb, ion transfer tube temperature 350 °C, and vaporizer temperature 250 °C. Acquired data were processed using TraceFinder software (version 3.1, Thermo Fisher Scientific, USA).

3.16S rRNA gene sequencing

Fecal microbial DNA was extracted using the Fecal DNA Extraction Kit (Tiangen Biotech, Cat#DP328-02, China) and integrity was verified by 1% agarose gel electrophoresis. The V3–V4 hypervariable regions of the 16S rRNA gene were amplified through PCR. The amplified PCR products were then sequenced on Illumina MiSeq platforms (Majorbio Bio-Pharm Technology Co., China) at equimolar concentrations, following the manufacturer's operating manual.

Bioinformatic processing was conducted on the Majorbio Cloud Platform. Alpha diversity was evaluated using Mothur (version 1.30.2) (1), while beta diversity was assessed using the Bray-Curtis dissimilarity matrix. Taxonomic profiles generated in QIIME (version 1.9.1) were visualized using: partial least squares discriminant analysis (PLS-DA) models for intergroup discrimination at the operational taxonomic unit (OTU) level, stacked bar plots (genus composition) constructed with ggplot2 in R language tools (version 3.3.1),

heatmaps using the vegan package, and differential taxa identification employed linear discriminant analysis effect size (LEfSe) with a linear discriminant analysis (LDA) score > 3 and $P < 0.05$.

4.Murine experimental models

4.1 Acute-on-Chronic Liver Failure (ACLF) mice with abdominal bacterial infection

The ACLF model was established using a modified protocol from Bin Gao et al. (2). Mice were randomized into 2 groups: control group and ACLF group. The ACLF mice underwent three phases of intervention: 1) chronic liver insult was induced by intraperitoneal (i.p.) injection of 0.2 mL/kg CCl_4 twice a week for 8 weeks; 2) acute liver insult was induced 3 days post-chronic insult by a single i.p. dose of 0.4 mL/kg CCl_4; 3) bacterial infection was induced 3 days after the acute liver insult via cecal ligation and puncture (CLP). The control mice underwent chronic liver injury, acute liver insult, and sham operation.

4.2 Cirrhosis mouse model

Mice underwent two phases of intervention: 1) chronic liver insult (i.p. 0.2 mL/kg CCl_4 twice a week for 8 weeks); 2) acute liver insult (i.p. 0.4 mL/kg CCl_4 , once) was induced 3 days post-chronic insult. This model was used to assess the effect of panose on liver injury before the mice progressed to infection-associated ACLF.

4.3 ACLF mice with pulmonary bacterial infection

Mice underwent three phases of intervention: 1) chronic liver insult (i.p. 0.2 mL/kg CCl_4 twice a week for 8 weeks); 2) acute liver insult (i.p. 0.4 mL/kg CCl_4 , once) was induced 3 days post-chronic insult; 3) bacterial infection was induced 3 days after the acute liver insult via intranasal administration of 1×10^{10} colony-forming unit (CFU) of *Klebsiella pneumoniae* (*K. P.*, ATCC BAA-1902). The control mice underwent chronic liver injury, acute liver insult, and an equivalent amount of phosphate buffered saline (PBS) via the same route.

4.4 Mice with acute polybacterial infection

The CLP model was established according to our prior research (3). Briefly, the cecum of mice was ligated with 4.0 silk thread, punctured bilaterally with an 18 G needle to expel some contents, and the skin was sutured. Sham surgery omitted cecal ligation and puncture. Mice then received a subcutaneous normal saline injection for resuscitation (1 mL/20 g body weight).

4.5 Mice with acute monobacterial infection

Mice were given a single i.p. injection of 5×10^8 CFU of *Escherichia coli* (*E. coli,* ATCC 25922) to generate an acute monobacterial infection. The control group of mice received an equal volume of PBS via the same route.

4.6 Advanced liver disease mice with ascites

Ascites model was established using a modified protocol from Nautiyal N et al. (4). Briefly, mice underwent two phases of intervention: 1) chronic liver insult (i.p. 0.2 mL/kg CCl_4 twice a week for 5 weeks and i.p. 0.5 mL/kg CCl_4 twice a week for 7 weeks); 2) acute liver insult (i.p. 1 mL/kg CCl_4, once) was induced 3 days post-chronic insult. The control mice underwent chronic liver injury, acute liver insult, and an equivalent amount of olive oil via the same route. Visible ascites developed 3 days after the acute liver insult.

5.Agent interventions in murine models

5.1 Agent interventions in ACLF mice and acute infection mice

To assess the effects of panose, ACLF mice or acute polybacterial-infected mice were randomly divided into 4 groups: Sham + PBS, Sham + Panose, CLP + PBS, and CLP + Panose. The acute monobacterial-infected mice were also randomly divided into 4 groups: PBS, Panose, *E. coli* + PBS, and *E. coli* + Panose. Panose (20 mg/kg) or PBS was gavaged for 3 consecutive days before bacterial infection.

To elucidate that panose protects intestinal barrier by inhibiting ROS, ACLF mice or acute infection mice were randomly divided into 4 groups: Sham + PBS, CLP + PBS, CLP + NAC, and CLP + NAC + Panose. Panose (20 mg/kg) was gavaged for 3 consecutive days before bacterial infection, and N-acetylcysteine (783 mg/kg) was administered 1 h before infection.

To evaluate the impact of a sequential treatment strategy involving panose and norfloxacin, ACLF mice were randomly divided into 8 groups: Pre-PBS + Sham, Pre-Panose + Sham, Sham + Post-Norfloxacin, Pre-Panose + Sham + Post-Norfloxacin, Pre-PBS + CLP, Pre-Panose + CLP, CLP + Post-Norfloxacin, and Pre-Panose + CLP + Post-Norfloxacin. Panose (20 mg/kg) was gavaged for 3 consecutive days before infection, whereas norfloxacin (6.67 mg/kg) was gavaged at 2 h, 14 h, and 26 h post-infection.

To evaluate the impact of a pre-infection combined administration strategy involving panose and norfloxacin, ACLF mice were randomly divided into 8 groups: Pre-PBS + Sham, Pre-Panose + Sham, Pre-Norfloxacin + Sham, Pre-Panose + Pre-Norfloxacin + Sham, Pre-PBS + CLP, Pre-Panose + CLP, Pre-Norfloxacin + CLP, and Pre-Panose + Pre-Norfloxacin + CLP. Panose (20 mg/kg) and norfloxacin (6.67 mg/kg) were gavaged for 3 consecutive days before infection. The norfloxacin dosage was determined based on a single administration of 400 mg for individuals (estimated at 60 kg) (5).

To assess the effects of isomalto-oligosaccharide (IMO), ACLF mice or acute infection mice were randomly divided into 4 groups: Sham + PBS, Sham + IMO, CLP + PBS, and CLP + IMO. IMO was gavaged for 3 consecutive days before infection at doses of 20, 50, and 100 mg/kg for the survival trial in acute infection mice, whereas 20 mg/kg was used for sample analysis in both ACLF mice and acute infection mice.

To evaluate the key components of IMO, each component (panose, isomaltotriose, isomaltotetraose, isomaltose, and IMO) was gavaged at 20 mg/kg for 3 consecutive days before infection.

ACLF mice were monitored for survival up to 10 days post-infection, while acute infection mice were monitored for survival up to 3 days post-infection. All samples were obtained 12 h post-bacterial infection for examination.

5.2 Agent interventions in mice with cirrhosis and ascites

To assess the effect of panose on liver cirrhosis, cirrhotic mice were randomly divided into 2 groups: PBS and Panose. Panose (20 mg/kg) or PBS was gavaged for 3 consecutive days starting 24 h after the acute liver insult.

To investigate the effect of panose on advanced liver disease mice with ascites, mice were randomly divided into 2 groups: PBS and Panose. Panose (20 mg/kg) or PBS was gavaged for 3 consecutive days starting at 72 h after the acute liver insult.

All samples were collected 12 h post-final treatment for analysis.

For a detailed list of agent catalog numbers, please refer to Supplementary Table 3.

6.Bacterial experiment

6.1 Whole-genome sequencing experiment

The employed *M. funiformis* was flash-frozen in liquid nitrogen. Genomic DNA was extracted, quality-checked, and sequenced on the DNBSEQ platform at BGI Genomics (Shenzhen, China). The average nucleotide identity (ANI) with closely related genomes was calculated using FastANI (version 2.0) against the NCBI RefSeq database.

6.2 Bacterial gene editing strategies

The sequence of the 4-alpha-glucanotransferase (4αGT, encoded by *malQ*) was identified from the whole-genome sequencing of *M. funiformis*. To knockout (KO) *malQ,* homologous recombination was employed. Approximately 1.5 kbp homology arms flanking *malQ* were cloned on either side of the kanamycin resistance (*KanR*) gene in the pUC57 vector. Recombinant plasmids were isolated using the Endo-free Plasmid Mini Kit II (Omega, Cat#D6950-01, USA). *M. funiformis* cells were collected at an OD_{600} of 0.6, resuspended to 1×10^{10} CFU in 10% glycerol, and electroporated with the recombinant plasmids at 25 μF, 200 Ω, and 1.8 kV for 5 ms using a Gene Pulser (BioRad, USA). After electroporation, culture media were quickly added to promote recovery. The *malQ* gene KO strain with the *KanR* gene was selected on blood agar with 50 μg/mL kanamycin and

confirmed by PCR with specific primers:

malQ-F: ATGATTTTTCATGATTCGCAAAATC,

malQ-R: CTATTATATTTTTCTACTAAGGAACAC.

KanR-F: TGGAGAGGCTATTCGGCTATGAC,

KanR-R: GCCGCCAAGCTCTTCAGCAATAT.

6.3 Bacterial growth curves

Wild-type (WT) and *malQ* KO *M. funiformis* strains were cultured in brain-heart infusion broth (BHI) medium at 37 °C for 48 h under anaerobic conditions. Bacterial proliferation was monitored (OD_{600}) at 12 h intervals over 48 h.

6.4 In vitro panose biosynthesis evaluation

To assess the capacity of *M. funiformis* to produce panose from pullulan *in vitro*, 4 groups were established: BHI medium, *M. funiformis* (2 × 10^9 CFU), Pullulan (100 mg/mL), and *M. funiformis* + Pullulan.

To evaluate the capacity of *malQ* KO strain of *M. funiformis* to produce panose from pullulan *in vitro*, 4 groups were established: BHI medium, Pullulan (100 mg/mL), WT (2 × 10^9 CFU) + Pullulan, and KO + Pullulan.

All groups were incubated at 37 °C for 48 h under anaerobic conditions. Post-incubation, supernatants were collected by centrifugation (4 °C, 3,000 g, 10 min) and panose was quantified by LC-MS/MS.

6.5 In vivo panose biosynthesis evaluation

Cirrhosis mice (underwent chronic and acute insults) were randomly divided into 3 groups: PBS, WT, and KO. The WT or *malQ* KO *M. funiformis* strain (1 × 10^9 CFU/day) was gavaged for 3 consecutive days starting 24 h after the acute insult. The control group of mice received an equal volume of PBS via the same route. Fecal samples collected 12 h post-final gavage were snap-frozen in liquid nitrogen and stored at -80 °C until analysis.

6.6 Assessment of M. funiformis' impact on ACLF mice

ACLF mice were randomly divided into 4 groups: Sham + PBS, CLP + PBS, CLP + WT, and CLP + KO. The WT or *malQ* KO *M. funiformis* strain (1 × 10^9 CFU/day) was gavaged for 3 consecutive days before infection. The control group of mice received an equal volume of PBS via the same route. Samples were collected at 12 h post-infection for analysis.

6.7 Microbial burden quantification

Intravenous blood and peritoneal lavage fluid (PLF) were aseptically collected from the mouse model 12 h post-infection. Samples were serially diluted with PBS and inoculated onto Columbia agar plates (Huankai, Cat#CP0160, Guangzhou, and Bio-Caring, Cat#P0901, Jiangmen, China), with one aliquot incubated under aerobic conditions and another under anaerobic conditions at 37 °C overnight. CFU were measured to assess microbial load.

6.8 Panose antimicrobial activity assay

To evaluate the direct impact of panose on bacterial proliferation, *E. coli* (1 × 10^5 CFU) was co-cultured in Luria-Bertani (LB) broth (Solarbio, Cat#L1010, China) containing different concentrations of panose (0, 50, 100, 200, 300, 400, 500, and 1,000 μM) at 37 °C under aerobic conditions for 12 h. Following incubation, bacterial samples were serially diluted with PBS and plated on LB agar (Solarbio, Cat#L1015, China). CFU were assessed after overnight incubation at 37 °C under aerobic conditions.

6.9 Phagocytosis and bacterial killing assays

Mice-derived bone marrow macrophages or neutrophils were isolated as previously described (3, 6). Cells were seeded in 12-well plates (2 × 10^5 cells/well) and pre-treated with 50 μM panose or PBS for 12 h.

To stimulate bacterial phagocytosis, cells were infected with *E. coli* (MOI 60:1 for

macrophages, 100:1 for neutrophils) in serum-free medium (37 °C, 5% CO_2, 45 min). After incubation, macrophages were washed with PBS containing 0.5 μg/mL gentamicin (Solarbio, Cat#L1312, China) and incubated with PBS containing 50 μg/mL gentamicin for 10 min to eliminate extracellular bacteria. Neutrophils were washed with PBS containing 0.5 μg/mL gentamicin and incubated with PBS containing 50 μg/mL gentamicin for 30 min to remove extracellular bacteria.

To evaluate phagocytosis, the cells after removing extracellular bacteria, were lysed with Triton X-100 (Solarbio, Cat#T8200, China) on ice (0.5% for macrophages, 0.1% for neutrophils) for 10 min to release intracellular bacteria. The cell lysate was serially diluted and inoculated onto LB agar to determine the number of bacteria engulfed (T1).

To evaluate bactericidal activity, the cells after removing extracellular bacteria were incubated in a medium containing 0.5 μg/mL gentamicin for 1 h at 37 °C under aerobic conditions. After incubation, the cells were washed with PBS and lysed with Triton X-100 on ice (0.5% for macrophages, 0.1% for neutrophils) for 10 min to liberate bacteria. The cell lysate was serially diluted and inoculated onto LB agar to assess the residual intracellular bacteria (T2). The percentage of bacteria killed was calculated using the formula [(count at T1 - count at T2) / (count at T1)] × 100% (6).

7.Sample detection

7.1 Biochemical analysis

Blood samples were centrifuged (3,500 rpm, 15 min, 4 °C) to separate plasma. Serum was collected after allowing the blood samples to clot at room temperature for 30 min, followed by centrifugation under identical conditions. An automatic analyzer (Olympus Company, Japan) was used to measure the concentrations of alanine aminotransferase (ALT), aspartate aminotransferase (AST), alkaline phosphatase (ALP), total bilirubin (TBIL), direct bilirubin (DBIL), and serum albumin, following the manufacturer's instructions.

7.2 Volume assessment of ascites

The *ascites* was extracted from the peritoneal cavity with a syringe, and the volume was recorded. The serum-ascites albumin gradient (SAAG) was calculated as follows: SAAG (g/dL) = [serum albumin] - [ascitic albumin].

7.3 Proportion of neutrophils and macrophages

Cell pellets were collected from PLF samples and resuspended in pre-chilled flow cytometry labeling buffer (eBioscience, Cat#00-4222-57, USA) after centrifugation (1,000 rpm, 10 min, 4 °C). The samples were incubated with fluorescently labeled flow cytometry antibodies (PE-F4/80, FITC-CD11B, and APC-cy7-LY6G) for 30 min at 4 °C in the dark. After staining, the cells were washed with PBS and resuspended before analysis using a flow cytometer (FACS Calibur, BD, USA) to identify neutrophils ($CD11B^{+}$ $LY6G^{+}$) and macrophages ($CD11B^{+}$ $F4/80^{+}$). Data were analyzed with FlowJo software (version 10, FlowJo, LLC). Antibody details are provided in Supplemental Table 4.

7.4 Histology, immunofluorescence, and microscopy

Liver hematoxylin & eosin (H&E) staining. H&E staining was performed on paraffin-embedded left liver slices (5 μm). Five or ten random areas of each section were quantified according to the five parameters (0-4 scale): interstitial edema, sinusoidal congestion, hepatocellular necrosis, hepatocellular vacuolization and leukocyte infiltration (7).

Sirius Red staining. The experiment was conducted on liver sections to evaluate collagen deposition using the modified Sirius Red Stain Kit (Solarbio, Cat#G1472, China) according to the manufacturer's instructions. The positive staining area was quantified using ten random fields of each slide with the assistance of ImageJ software (version 1.53).

TUNEL staining. Ileum sections were stained using an Apoptosis Detection Kit (KeyGEN Biotech, Cat#KGA7063, China) according to the manufacturer's instructions.

Observation of tight junctions. Ileum tissue was fixed in 2.5% glutaraldehyde and post-fixed in 1% osmium tetroxide for structural preservation. The tissues were then

dehydrated in a graded series of ethanol and embedded in paraffin for sectioning. Ultrathin sections were cut from the paraffin blocks and mounted on copper grids. After staining, the sections were visualized using transmission electron microscopy (TEM, JEM-1200; Jeol Ltd., Tokyo, Japan) at 60 kV, with representative images captured for analysis.

Immunofluorescence staining of the occludin protein. Paraffin-embedded ileum sections were deparaffinized with xylene and then rehydrated with ethanol dilutions and PBS. Antigen unmasking was performed using Tris-EDTA buffer followed by permeabilization and blocking. Sections were incubated overnight with occludin antibody (1:100), washed, and then incubated with a secondary antibody (1:1,000). Nuclei were stained with DAPI (1:1,000) before mounting. Images were captured with a fluorescence microscope (Carl Zeiss, LSM 880, Germany). Antibody details are provided in Supplemental Table 4.

Tissue ROS staining. Fresh ileum tissue was frozen in liquid nitrogen, embedded in an optimal cutting temperature compound (OCT, SAKURA, Cat#4583, Japan), and sectioned into 8 μm slices using a cryostat (Leica CM-3050S, Heerbrugg, Switzerland) at -20 °C. To detect ROS, the slides were incubated for 15 min with 30 μM dihydroethidium (DHE, Invitrogen, Cat#D23806, USA) in the dark. After incubation, the slides were washed three times with PBS to remove unbound DHE. To stain the cell nucleus, the slides were then incubated with 10 μg/mL DAPI (Beyotime, Cat#C1002, China) for 5 min at room temperature in the dark. The slides were then washed three times with PBS and images were captured with a fluorescence microscope (Carl Zeiss, LSM 880, Germany). The mean fluorescence intensity (MFI) of ROS was quantified using ImageJ software (version 1.53) to assess the level of oxidative stress in the tissue sections.

7.5 Bacterial translocation assay

The *E. coli* BL21 (DE3) strain (Vazyme, Cat#C504-02/03, China) was transformed with a GFP-encoding plasmid to construct *GFP-E. coli.* Transformation involved incubating 100 μL of bacterial suspension with 1 μL of plasmid DNA (30 min ice bath), followed by a 90 sec heat-shock at 42 °C and a 2 min re-ice bath. Transformed cells were recovered in

antibiotic-free LB medium (37 °C, 1 h) before plating on ampicillin-containing agar (50 μg/mL) for overnight incubation. Single colonies were cultured in LB medium with ampicillin to an OD_{600} of 0.6, then induced with 1 mM isopropyl β-D-1-thiogalactopyranoside (IPTG, Solarbio, Cat#I1020, China) for 4 h of GFP expression. Bacterial pellets were obtained by centrifugation (4 °C, 4,000 rpm, 10 min) and confirmed by fluorescence microscopy.

1 × 10^9 CFU *GFP-E. coli* was orally administered 3 h after sham or CLP surgery, and liver samples were collected 12 h post-surgery. The left liver lobes were embedded in OCT, sliced and fixed in methanol overnight. After air-drying, slices were placed in an anti-fade mounting medium with DAPI (Beyotime, Cat#P0131, China) and imaged at 40 × magnification using a fluorescence microscope (Carl Zeiss, LSM 880, Germany). Fluorescent signals were manually quantified in 5 or 10 random fields in each section.

7.6 Lipopolysaccharides (LPS) detection

Blood was obtained from the inferior vena cava of mice by using an anticoagulant tube. Plasma was collected after centrifugation at 3,000 g for 30 min at 4 °C. The LPS concentrations were quantified according to the protocol of the commercial LPS ELISA Kit (LanpaiBIO, Cat#hj-C9120, China).

8.Cell experiments

8.1 Cell treatment and transfection

To assess the effect of panose on cell viability, Mode-K cells were treated with panose at concentrations ranging from 0 to 100 μM and incubated at 37 °C for 12 h. Cell viability was assessed using the Cell Counting Kit-8 (CCK-8, Dojindo, Cat#CK04, Japan).

To test the protective effect of panose against intracellular oxidative stress, Mode-K cells were pre-treated with 50 μM panose or PBS for 2 h before being exposed to 0.003% hydrogen peroxide (H_2O_2, Sigma-Aldrich, Cat#88597, USA) for 12 h.

To decrease the expression of xCT protein, four small interfering RNAs (siRNAs) targeting

xCT (si-xCT, #1-4) and a negative control siRNA (si-NC) were obtained from GenePharma (Suzhou, China) and separately transfected into Mode-K cells. The si-xCT with statistically significant transfection efficiency was selected for subsequent experiments. The interference sequences for xCT are listed in Supplementary Table 5. To increase xCT protein expression, the xCT overexpression plasmid (ov-xCT) and negative control plasmid (ov-NC) were obtained from Genechem (Shanghai, China). All transfection experiments were conducted using lipofectamine 2000 reagent (Invitrogen, Cat#11668019, USA) following the manufacturer's instructions. Cells were eligible for subsequent experiments 48 h after transfection.

8.2 Assessment of oxidative stress

The activity of superoxide dismutase (SOD, Beyotime, Cat#S0101M, China), malondialdehyde (MDA, Beyotime, Cat#S0131M, China), and glutathione (GSH, Nanjing Jiancheng Bioengineering Institute, Cat#A061-1-2, China) levels were measured in ileum tissue or Mode-K cells according to the manufacturer's instructions. Values were normalized by protein or tissue weight.

To identify intracellular ROS, Mode-K cells were harvested, washed with cold PBS, and stained with a freshly prepared high-sensitivity photooxidation-resistant dye, 2',7'-Dichlorodihydrofluorescein diacetate (DCFH-DA, 1:1,000, Dojindo, Cat#R253, Japan). After 30 min of incubation at 37 °C in the dark, the cells were washed with Hank's balanced salt solution (HBSS, Gibco, Cat#14175095, USA) and resuspended for measurement of MFI using a flow cytometer (FACS Calibur, BD, USA).

8.3 Separation of the lamina propria

The ileum tissue was longitudinally opened and washed with cold PBS to remove fat tissue and lymph nodes. The tissue was then cut into small pieces and placed in PBS containing 3% fetal bovine serum (FBS), 1 mM ethylenediaminetetraacetic acid (EDTA, Solarbio, Cat#E1170, China), and 1 mM dithiothreitol (DTT, Solarbio, Cat#D8220, China), then incubated for 20 min on a horizontal shaker at 37 °C, 250 rpm to separate the mucus

and epithelial layers. Subsequently, the tissue was washed twice with PBS and placed in RPMI 1640 medium (Gibco, Cat#C11875500BT, USA) containing 3% FBS, 0.2 mg/mL collagenase IV (Worthington, Cat#LS004189, USA), and 0.1 mg/mL DNase I (Solarbio, Cat#D8071, China) on a shaker at 37 °C, 250 rpm for 30 min digestion. After digestion, the cell pellet was collected by centrifugation at 400 g, 4 °C for 8 min. After removing red blood cells, the remaining cells were resuspended in PBS, filtered through a 70 μm cell strainer, and used for RT-qPCR.

8.4 Detection of amino acid levels

The cystine uptake was conducted using two methods: 1) Mode-K cells were collected and incubated with cystine-free DMEM (Gibco, Cat#21013024, USA) containing 5 μM FITC-cystine (Sigma-Aldrich, Cat#SCT047, USA) for 30 min at 37 °C in the dark. After washing with PBS, cells were analyzed using a flow cytometer (FACS Calibur, BD, USA). 2) Mode-K cells were harvested and processed using the Cystine Uptake Assay Kit (Dojindo, Cat#UP05, Japan). The fluorescence intensity was measured at Ex/Em: 490/535 nm.

To detect glutamate efflux, Mode-K cells were harvested and centrifuged at 12,000 rpm at 4 °C for 15 min to separate the supernatant and pellet. The supernatant was used to measure the glutamate excretion levels following the instructions of the Glutamate Measurement Kit (Nanjing Jiancheng Bioengineering Institute, Cat#A074-1-1, China). The pellet was used to quantify the protein concentration for standardizing the glutamate levels.

To detect cysteine levels, Mode-K cells were harvested and centrifuged at 12,000 rpm at 4 °C for 15 min to collect the cell pellet. Cysteine levels were detected by using a Cysteine Content Test Kit (Nanjing Jiancheng Bioengineering Institute, Cat#A126-1-1, China) according to the manufacturer's protocol, and normalized by protein concentration.

8.5 Drug affinity responsive target stability (DARTS) assay

Mode-K cells were lysed with 500 μL of M-PER lysis buffer (Thermo Scientific, Cat#78501,

USA) containing protease and phosphatase inhibitors for 30 min on ice. After centrifugation at 18,000 g for 10 min at 4 °C, the supernatant was collected and adjusted to 5 mg/mL. The supernatant was divided into two tubes, one incubated with 50 μM panose and the other with PBS, for 2 h at room temperature. Pronase (Roche, Cat#11459643001, Switzerland) was added to the protein samples at a 1:500 (w/w) ratio for 30 min at room temperature. The reaction was terminated by adding loading buffer and heating at 95 °C for 5 min. Samples were analyzed by Western blotting.

8.6 Cellular thermal shift assay (CETSA)

Mode-K cells were harvested using RIPA lysate containing phosphatase and protease inhibitors. The lysate was rapidly frozen in liquid nitrogen for three cycles of 30 sec each. After centrifugation at 15,000 rpm for 15 min at 4 °C, the supernatant was divided into two tubes and treated with 50 μM panose or PBS for 2 h. Subsequently, 100 μL of each sample was aliquoted into 10 PCR tubes and heated at 37 °C, 41 °C, 44 °C, 47 °C, 50 °C, 53 °C, 56 °C, 59 °C, 63 °C and 67 °C for 3 min, then stored at room temperature for 3 min, and finally stored on ice. All samples were centrifuged at 15,000 rpm for 40 min at 4 °C. The protein supernatant was collected, mixed with loading buffer, heated at 95 °C for 5 min, and analyzed via Western blotting.

9.Molecular Docking

The crystal structure of xCT (PDB ID: 7EPZ, Chain B) was obtained from the RCSB Protein Data Bank and the panose ligand structure (CID: 94448) was retrieved from the PubChem database. The protein preparation was conducted in PyMOL (version 1.83) by eliminating water molecules and preserving only Chain B. AutoDockTools (version 1.5.7) was employed to add charges and hydrogen atoms to the protein, which was then set as a rigid body. The panose ligand was prepared through the assignment of Gasteiger charges and the identification of rotatable bonds using AutoDockTools (version 1.5.6). The xCT extracellular domain sequence from UniProt was mapped onto the xCT 3D structure using PyMOL. In AutoDockTools (version 1.5.6), a docking grid box was configured with the

following parameters: center_x = 145.649, center_y = 141.347, center_z = 145.631, size_x = 48.2222222222, size_y = 54.25, size_z = 36.1666666667. Molecular docking was conducted via AutoDock Vina (version 1.2.5), and the conformation exhibiting the lowest binding energy was chosen and visualized in PyMOL.

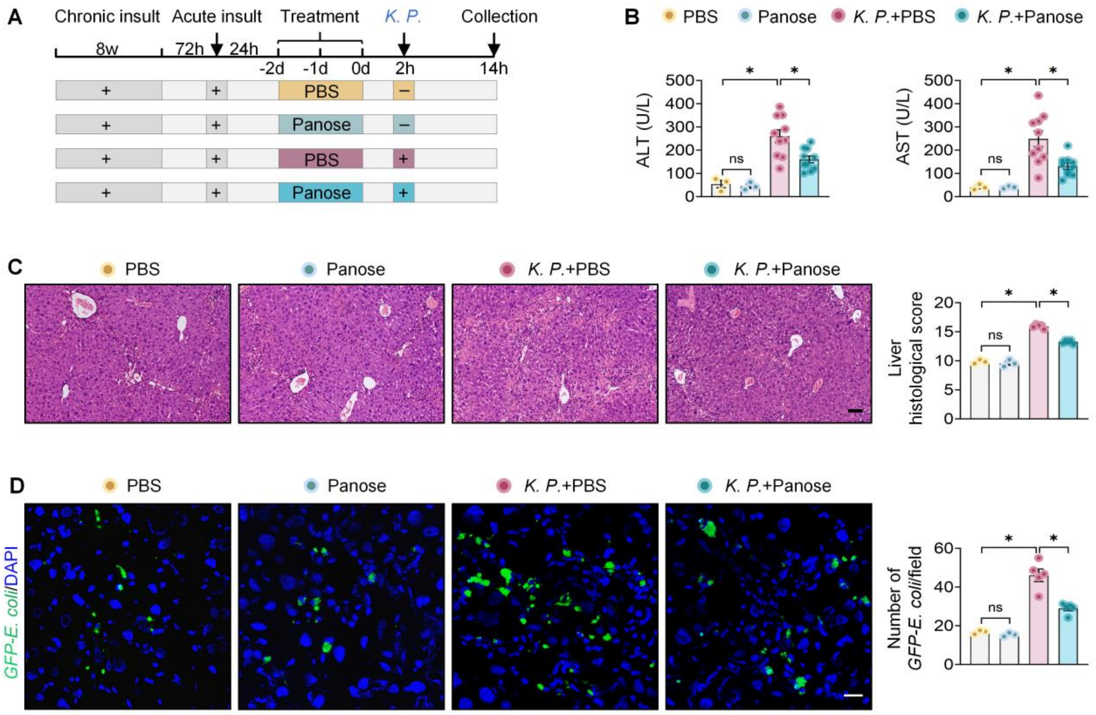

Supplementary Figure 1. Panose administration attenuates ACLF mice with pulmonary bacterial infection. (A) Schematic diagram of ACLF mice with pulmonary bacterial infection. Infection was induced by intranasal injection of 1 × 10^{10} CFU *K. P.*, and control mice were injected with an equal amount of PBS. Samples were collected at 12 h post-infection for subsequent analysis. **(B)** Plasma levels of ALT and AST (n = 3-10/group). **(C)** Representative images and quantification of H&E-stained liver sections. Scale bar: 100 μm (n = 3-5/group). **(D)** Representative images and quantification of *GFP-E. coli* fluorescence intensity in the liver of ACLF mice with pulmonary bacterial infection (green: *GFP-E. coli*; blue: DAPI-stained nuclei). Scale bar: 20 μm (n = 3-5/group). Data are presented as mean ± SEM. Statistical significance was determined by 1-way ANOVA with Bonferroni post-hoc test. $^{*}P < 0.05$. ns, non-significance. ACLF, acute-on-chronic liver failure; CFU, colony-forming unit; *K. P., Klebsiella pneumoniae;* PBS, phosphate-buffered saline; ALT, alanine aminotransferase; AST, aspartate aminotransferase; H&E, hematoxylin & eosin; *GFP-E. coli*, green fluorescent protein-labeled *Escherichia coli*.

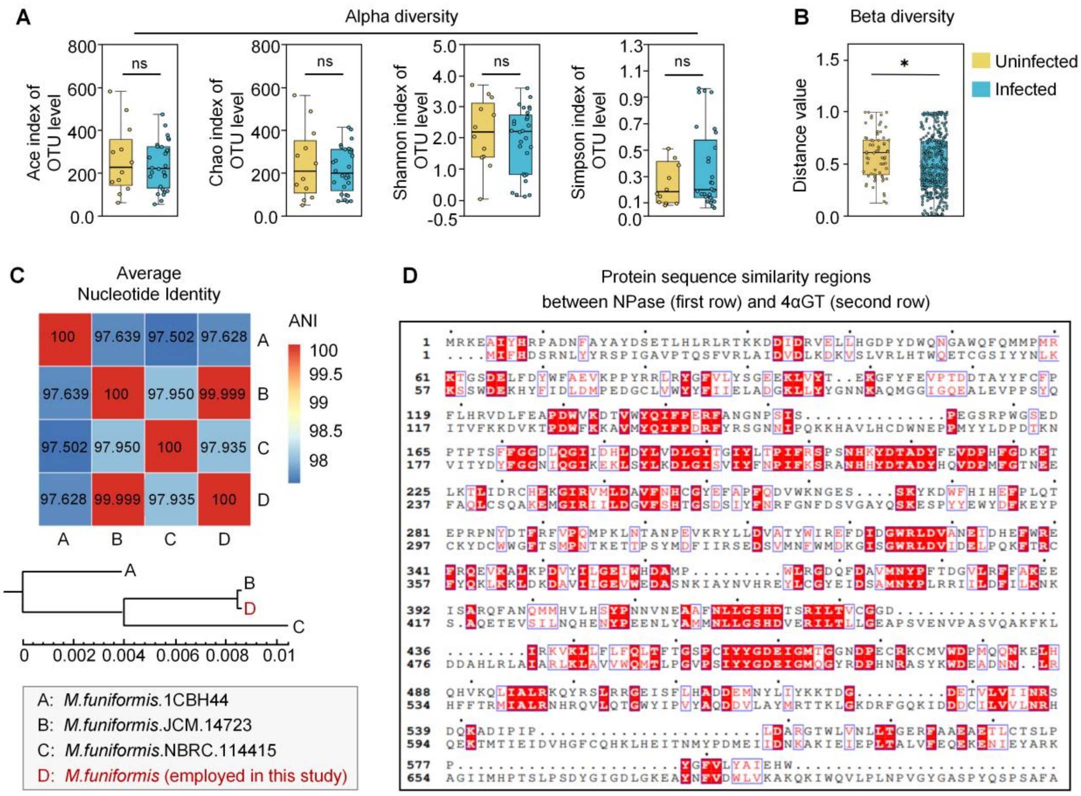

Supplementary Figure 2. Microbiome analysis and protein sequence similarity alignment. (A) Fecal microbiota alpha diversity was assessed using Ace, Chao, Shannon, and Simpson indexes (n = 12-28/group). **(B)** Beta diversity analysis plot of fecal microbiota. The vertical axis shows beta diversity distances between samples, with the box plot's central dot indicating the distance index between two samples. The uninfected group (n=12) has 66 dots in the box, while the infected group (n = 28) has 378 dots in the box. **(C)** ANI analysis of the sequenced (employed *M. funiformis* strain) and NCBI database strains (*M. funiformis* 1CBH44, JCM.14723, and NBRC.114415). Phylogenetic analysis based on whole genome SNPs showed that the employed *M. funiformis* strain was most related to *M. funiformis* JCM.14723. **(D)** The amino acid sequences of NPase (first row) and 4αGT (second row) were aligned by the ClustalW method and visualized with ESPript 3.0 (red box, white character: strict identity; red character: similarity in a group; blue frame: similarity across groups). Data are presented as median ± interquartile range **(A and B)**. Statistical significance was determined by Mann-Whitney U test **(A and B)**. $^{*}P < 0.05$. ns, non-significance. Ace, abundance-based coverage estimator; DC, decompensated cirrhosis; ANI, average nucleotide identity; *M. funiformis*, *Megamonas funiformis*; SNPs, single nucleotide polymorphisms; NPase, neopullulanase; 4αGT, 4α-glucanosyltransferase.

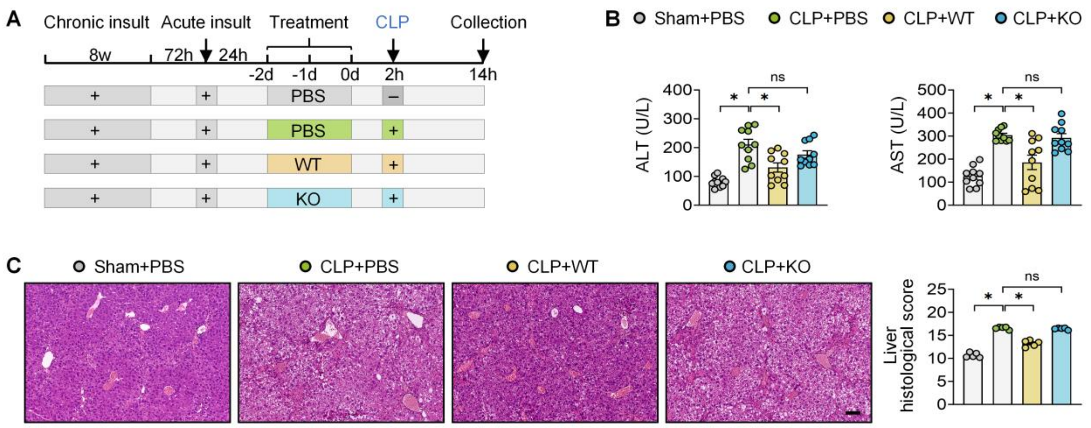

Supplementary Figure 3. The *malQ* KO strain of *M. funiformis* has no apparent protective effect on ACLF mice. (A) Schematic timeline of WT and *malQ* KO strains treatment in ACLF mice. Samples were collected at 12 h post-infection for subsequent analysis. **(B)** Plasma levels of ALT and AST (n = 10/group). **(C)** Representative images and quantification of H&E-stained liver sections. Scale bar: 100 μm (n = 5/group). Data are presented as mean ± SEM. Statistical significance was determined by 1-way ANOVA with Bonferroni post-hoc test. $^{*}P < 0.05$. ns, non-significance. *malQ* (encoding 4αGT), 4α-glucanosyltransferase; WT, wild-type; KO, knockout.

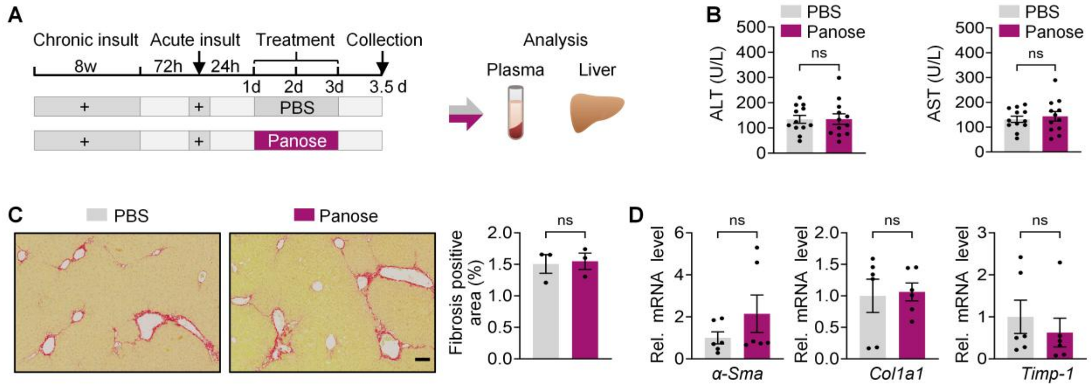

Supplementary Figure 4. Panose treatment has no substantial effect on liver injury during the pre-infection stage of ACLF. (A) Mice were subjected to chronic insult and acute insult. Samples were collected at 12 h after the final treatment for analysis. **(B)** Plasma levels of ALT and AST (n = 12/group). **(C)** Representative images and quantification of Sirius Red staining in liver sections. Scale bar: 100 μm (n = 3/group). **(D)** The mRNA levels of fibrosis markers (*α-Sma*, *Col1a1*, and *Timp1*) in liver tissue were determined by RT-qPCR (n = 6/group). Data are presented as mean ± SEM. Statistical significance was determined by Student's t-test **(B)** and Mann-Whitney U test **(C** and **D)**. ns, non-significance. *α-Sma,* α-smooth muscle actin; *Col1a1*, collagen type I alpha 1 chain; *Timp1*, tissue inhibitor of metalloproteinases 1.

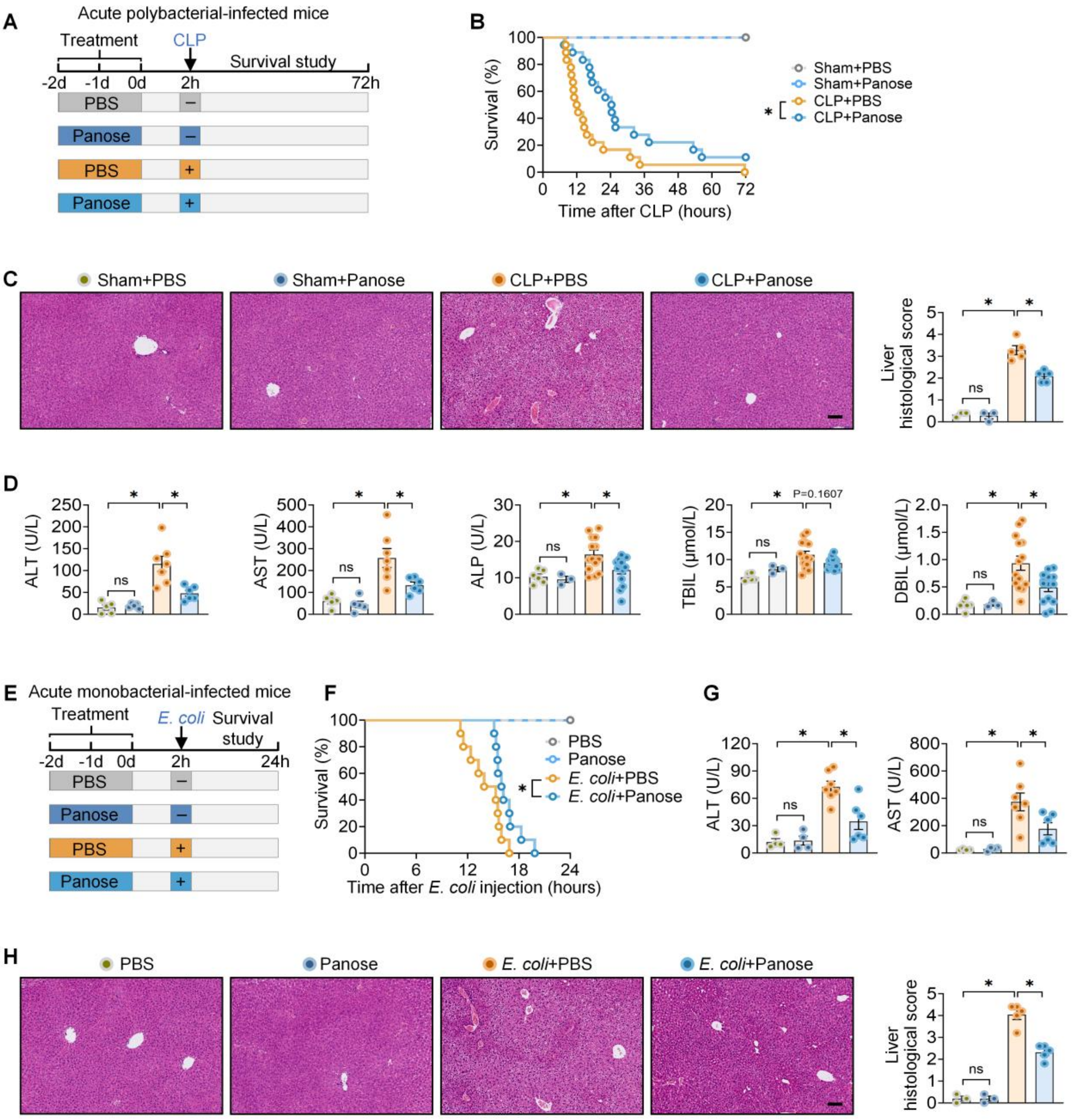

Supplementary Figure 5. Panose protects mice against acute polybacterial and monobacterial infections. (A) Schematic timeline of panose treatment and survival study in an acute polybacterial-infected mouse model. Infection was induced via CLP, while uninfected mice underwent a sham operation. **(B)** Kaplan-Meier survival curves for acute polybacterial-infected mice that were treated with PBS or panose (n = 5-18/group). **(C)** Representative images and quantification of H&E-stained liver sections from acute polybacterial-infected mice at 12 h post-infection. Scale bar: 100 μm (n = 3-5/group). **(D)** Plasma levels of ALT, AST, ALP, TBIL, and DBIL in acute polybacterial-infected mice at 12 h post-infection (n = 3-14/group). **(E)** Schematic timeline of panose treatment and survival study in an acute monobacterial-infected mouse model. Infection was induced by intraperitoneal injection of 5 × 10^8 CFU *E. coli*, while uninfected control mice received an equal volume of PBS. **(F)** Kaplan-Meier survival curves for acute monobacterial-infected mice that were treated with PBS or panose (n = 5-10/group). **(G)** Plasma levels of ALT and AST in acute monobacterial-infected mice at 12 h post-infection (n = 4-7/group). **(H)** Representative images and quantification of H&E-stained liver sections from acute monobacterial-infected mice at 12 h post-infection. Scale bar: 100 μm (n = 3-5/group).

Data are presented as mean ± SEM. Statistical significance was determined by Log-rank test **(B and F)** and 1-way ANOVA with Bonferroni post-hoc test (**C, D**, **G**, and **H**). $^{*}P < 0.05$. ns, non-significance. CLP, cecal ligation and puncture; ALP, alkaline phosphatase; TBIL, total bilirubin; DBIL, direct bilirubin.

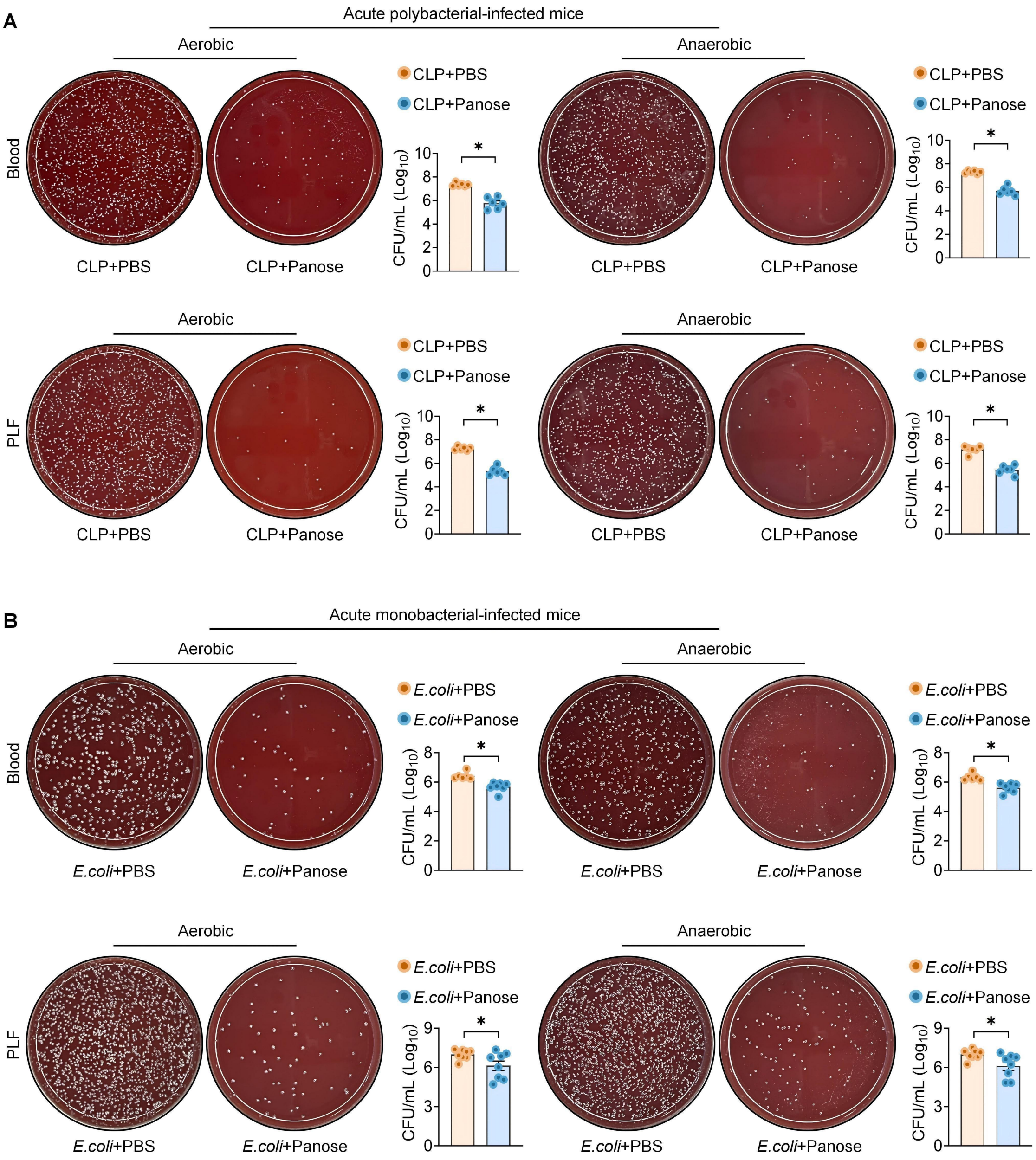

Supplementary Figure 6. Panose combats bacterial infection in murine models of acute infection. (A-B) Representative images and statistical plots of colony formation in peripheral blood and PLF samples after incubation in aerobic or anaerobic conditions. Samples were collected at 12 h post-infection from: **(A)** acute polybacterial-infected mice treated with PBS or panose (n = 6/group), and **(B)** acute monobacterial-infected mice treated with PBS or panose (n = 6-8/group). All CFU values were $\log_{10}$-transformed. Data are presented as mean ± SEM. Statistical analysis used Student's t-test except for the anaerobic PLF sample plot in **(A)** and the aerobic blood sample plot in **(B)**, which used the

Mann-Whitney U test. $^{*}P < 0.05$. PLF, peritoneal lavage fluid; CFU, colony-forming unit.

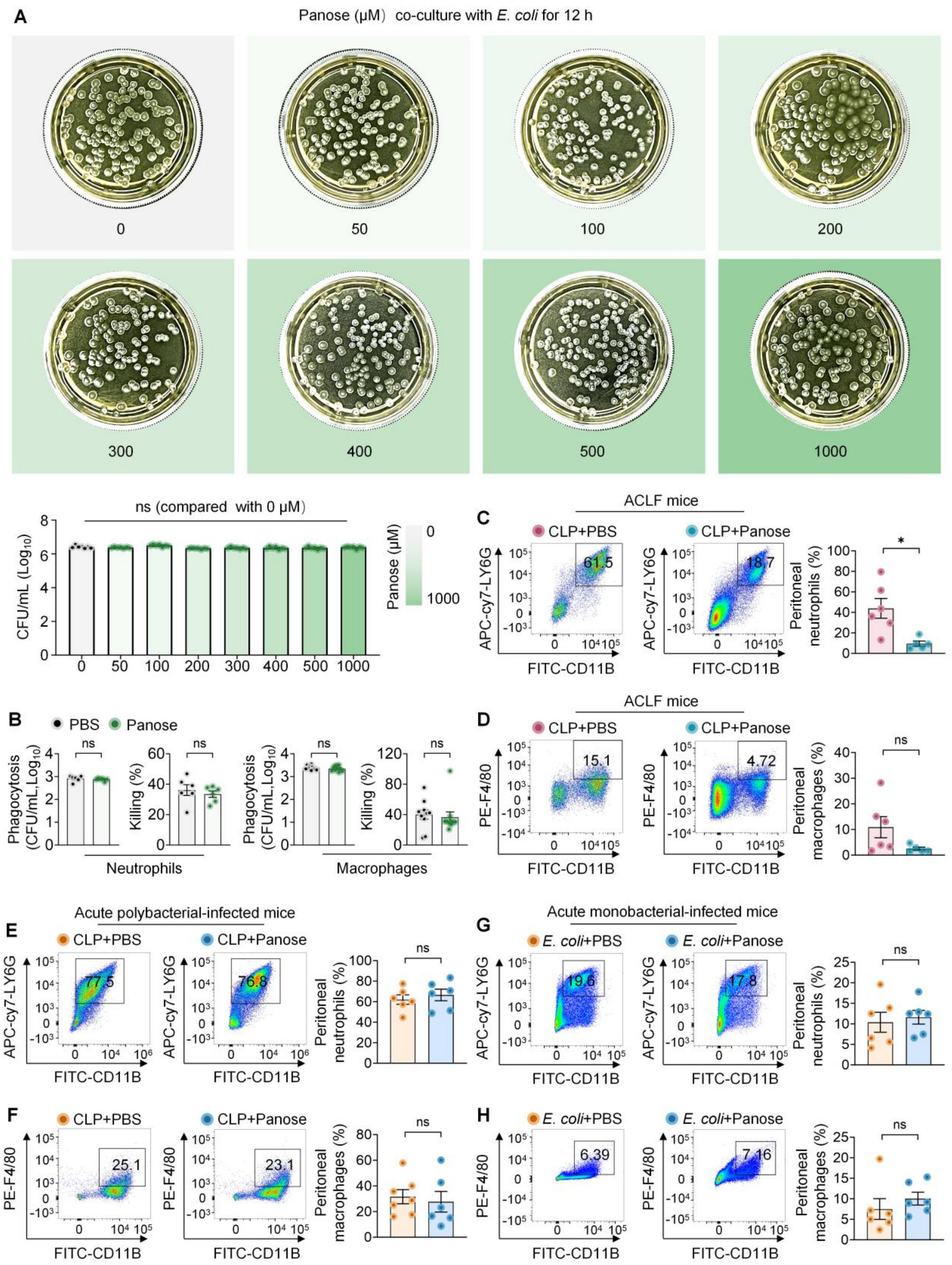

Supplementary Figure 7. Panose exhibits no bactericidal effect and does not substantially increase immune cell proportions or functions. (A) The growth of *E. coli* after 12 h of co-culture with varying panose doses (n = 7/group). **(B)** Evaluation of panose effects on phagocytic and bactericidal abilities in neutrophils and macrophages (n = 6-10/group). **(C-H)** Flow cytometric analysis of neutrophils ($CD11B^{+}$ $LY6G^{+}$) and macrophages ($CD11B^{+}$ $F4/80^{+}$) proportions in PLF from: **(C** and **D)** ACLF mice (n =

5-6/group), **(E** and **F)** acute polybacterial-infected mice (n = 6-7/group), and **(G** and **H)** acute monobacterial-infected mice (n = 6/group). Data are presented as mean ± SEM. Statistical significance was determined by 1-way ANOVA with Bonferroni post-hoc test **(A),** Mann-Whitney U test for macrophage bactericidal rate **(B)** and macrophage percentage **(H)**, and Student's t-test for the rest. $^{*}P$ < 0.05. ns, non-significance. PLF, peritoneal lavage fluid.

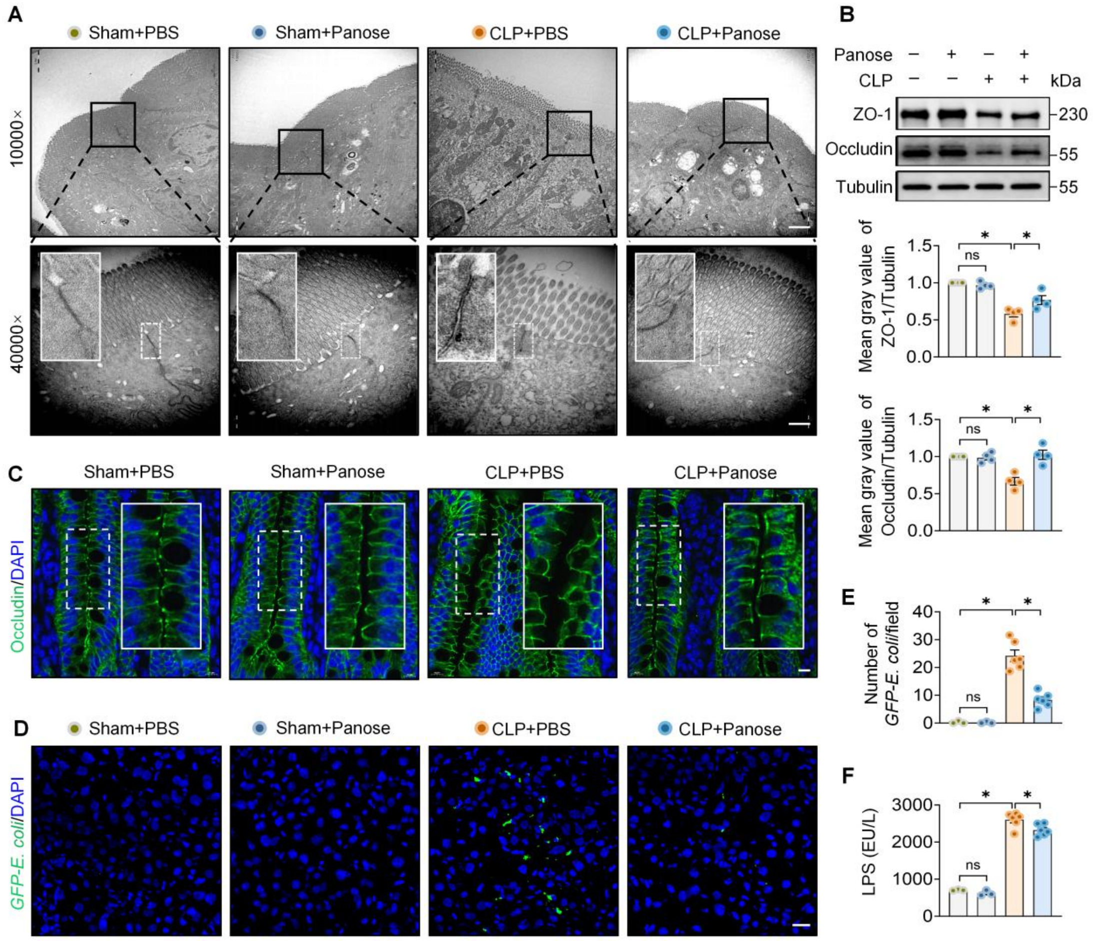

Supplementary Figure 8. Panose restores the intestinal barrier in acute polybacterial-infected mice. (A) Representative TEM images of ileum TJs in acute polybacterial-infected mice (10,000×, scale bar: 2 µm; 40,000×, scale bar: 500 nm). **(B)** Western blot analysis and quantification of ZO-1 and occludin protein expression in the ileum of acute polybacterial-infected mice (n = 4/group). **(C)** Representative immunofluorescence images of occludin in the ileum of acute polybacterial-infected mice (green: occludin; blue: DAPI-stained nuclei). Scale bar: 10 µm. **(D** and **E)** Representative images **(D)** and quantification **(E)** of *GFP-E. coli* fluorescence intensity in the liver of acute polybacterial-infected mice (green: *GFP-E. coli*; blue: DAPI-stained nuclei). Scale bar: 20 µm (n = 3-6/group). **(F)** LPS levels in the plasma of acute polybacterial-infected mice at 12 h post-infection (n = 3-6/group). Data are presented as mean ± SEM. Statistical significance was determined by 1-way ANOVA with Bonferroni post-hoc test. $^{*}P$ < 0.05. ns, non-significance. TEM, transmission electron microscopy; TJs, tight junctions; ZO-1,

zonula occludens-1; LPS, lipopolysaccharide.

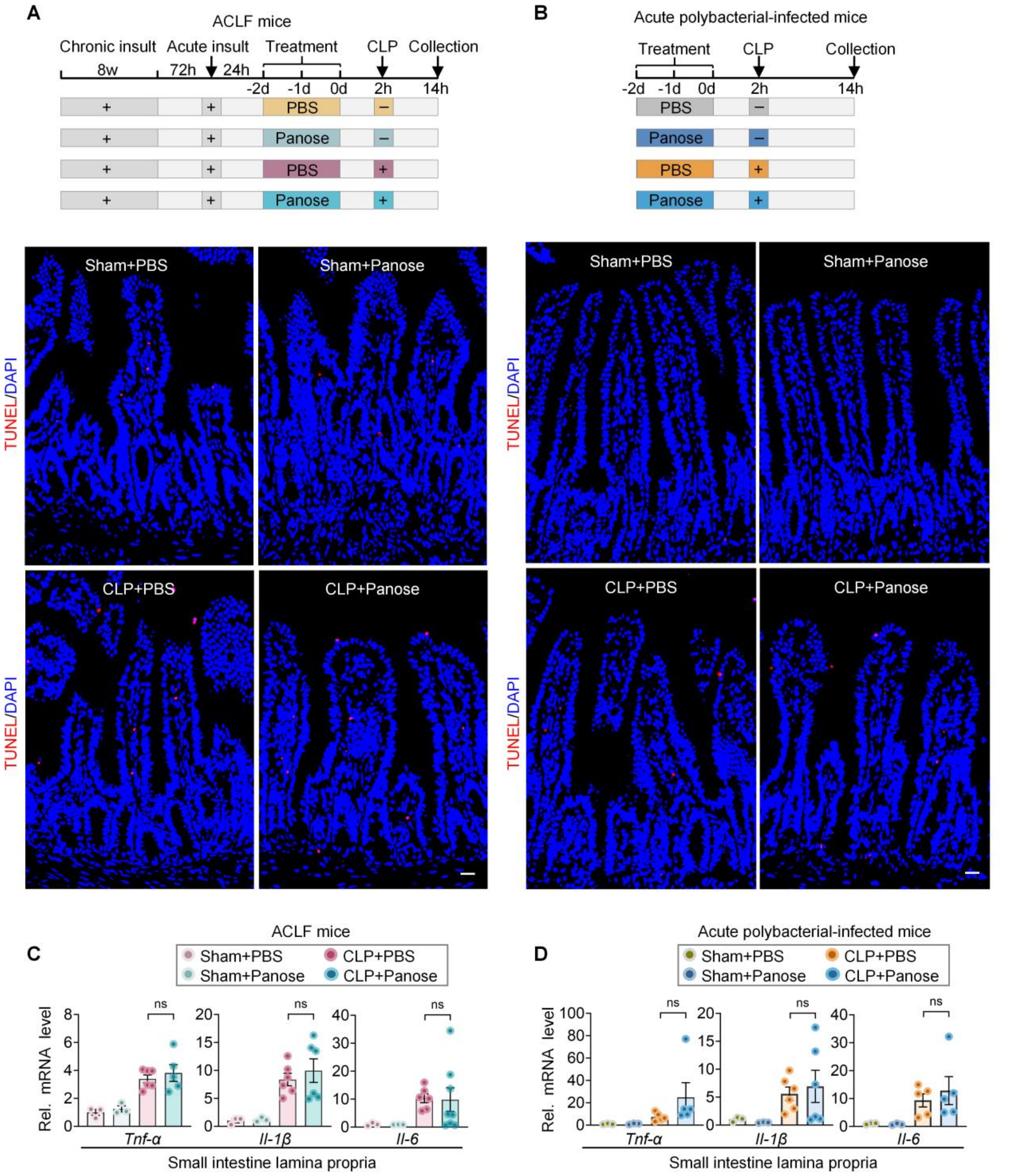

Supplementary Figure 9. Panose has limited effects on intestinal apoptosis and inflammation. (A and B) Representative TUNEL staining images of the ileum from **(A)** ACLF mice and **(B)** acute polybacterial-infected mice (red: TUNEL-positive cells; blue: DAPI-stained nuclei). Scale bar: 20 μm. **(C and D)** The mRNA levels of inflammation markers (*Tnf-α*, *Il-1β*, and *Il-6*) in the ileum lamina propria of **(C)** ACLF mice (n = 3-8/group) and **(D)** acute polybacterial-infected mice (n = 3-6/group) were determined by RT-qPCR. Data are presented as mean ± SEM. Statistical significance was determined by 1-way ANOVA with Bonferroni post-hoc test. $^{*}P < 0.05$. ns, non-significance. *Tnf-α*, tumor necrosis factor-alpha; *Il-1β*, interleukin-1 beta; *Il-6*, interleukin-6.

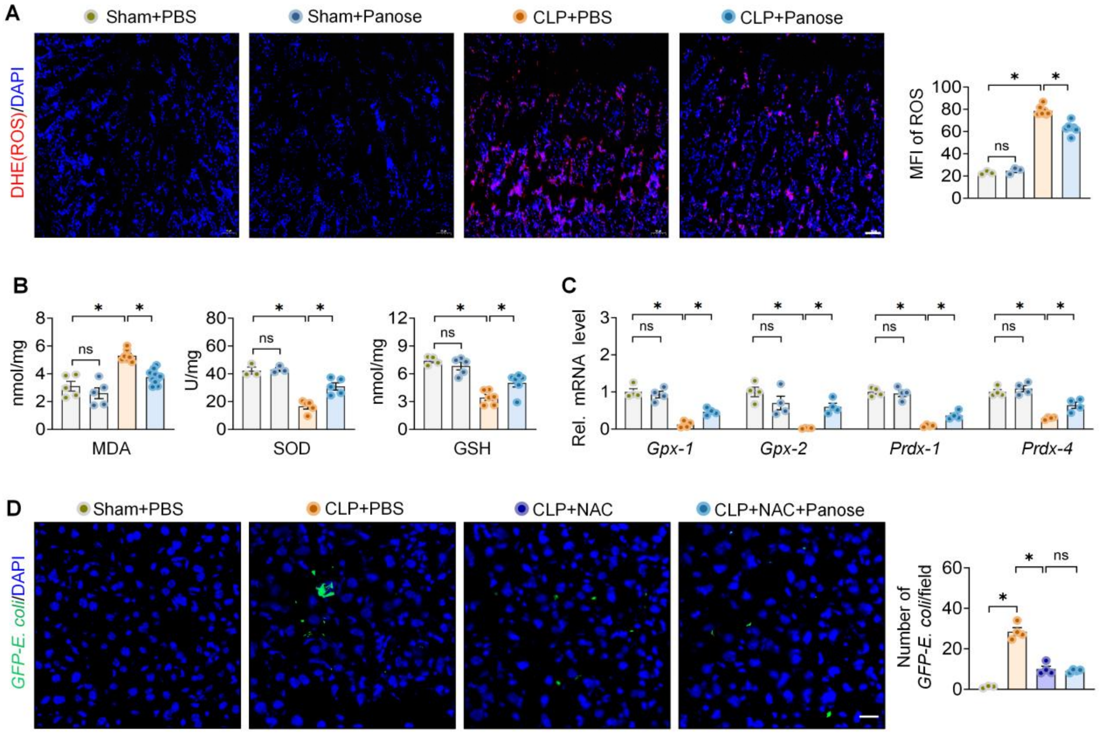

Supplementary Figure 10. Panose improves gut barrier dysfunction by reducing intestinal oxidative stress in acute polybacterial-infected mice. (A) ROS levels in ileum sections of acute polybacterial-infected mice were visualized with DHE staining and quantified using MFI (red: ROS; blue: DAPI-stained nuclei). Scale bar: 50 μm (n = 3-7/group). **(B)** MDA, SOD, and GSH levels in the ileum tissue of acute polybacterial-infected mice at 12 h post-infection (n = 3-10/group). **(C)** The mRNA levels of antioxidant markers (*Gpx-1, Gpx-2, Prdx-1*, and *Prdx-4*) in the ileum tissue of acute polybacterial-infected mice were determined by RT-qPCR (n = 4/group). **(D)** Representative images and quantification of *GFP-E. coli* fluorescence intensity in the liver of acute polybacterial-infected mice after ROS elimination with NAC (green: *GFP-E. coli*; blue: DAPI-stained nuclei). Scale bar: 20 μm (n = 3-4/group). Data are presented as mean ± SEM. Statistical significance was determined by 1-way ANOVA with Bonferroni post-hoc test. $^{*}P$ < 0.05. ns, non-significance. ROS, reactive oxygen species; DHE, dihydroethidium; MFI, mean fluorescence intensity; MDA, malondialdehyde; SOD, superoxide dismutase; GSH, glutathione; *Gpx-1,* glutathione peroxidase 1; *Gpx-2,* glutathione peroxidase 2; *Prdx-1*, peroxiredoxin 1; *Prdx-4,* peroxiredoxin 4; NAC, N-acetylcysteine.

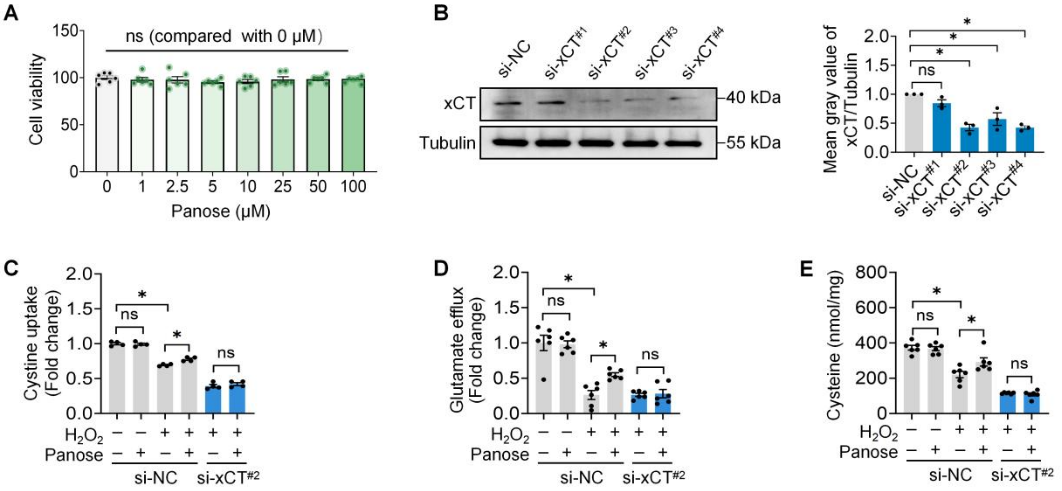

Supplementary Figure 11. Mode-K cell viability and xCT interference efficiency. (A) The impact of different panose concentrations on Mode-K cell viability was assessed using the CCK-8 assay (n = 6/group). **(B-E)** Mode-K cells transfected with si-NC or si-xCT were analyzed as follows: **(B)** Western blot analysis and quantification of xCT expression (n = 3/group). **(C)** Cystine uptake was measured with a fluorescent enzyme labeler at 490/535 nm (n = 4/group). **(D)** Glutamate efflux levels in the media (n = 6/group). **(E)** Intracellular cysteine levels (n = 6/group). Data are presented as mean ± SEM. Statistical significance was determined by 1-way ANOVA with Bonferroni post-hoc test. $^{*}P < 0.05$. ns, non-significance. xCT (known as SLC7A11), solute carrier family 7 member 11; CCK-8, cell counting kit-8; si-NC, negative control small interfering RNA; si-xCT, xCT-specific small interfering RNA.

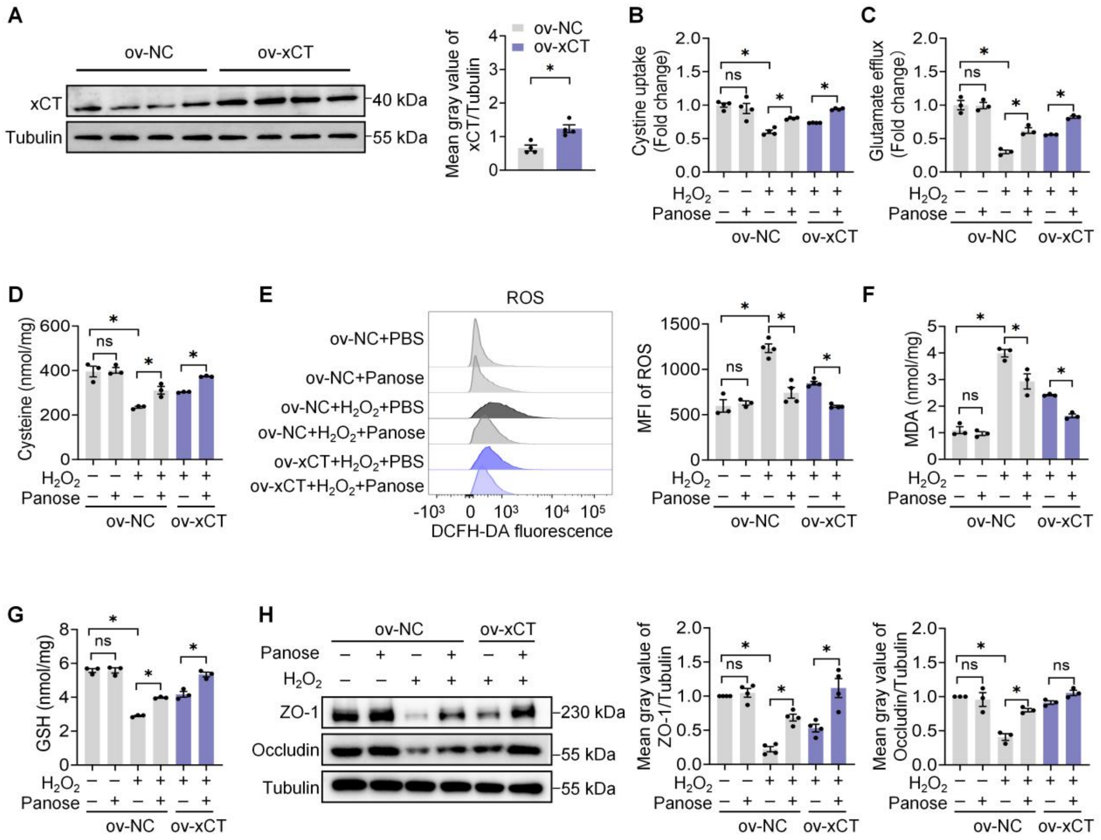

Supplementary Figure 12. Overexpression of xCT enhances panose's role in inhibiting oxidative stress. Mode-K cells transfected with ov-NC or ov-xCT plasmid were analyzed as follows: **(A)** Western blot analysis and quantification of xCT expression (n = 4/group). **(B)** Cystine uptake was measured with a fluorescent enzyme labeler at 490/535 nm (n = 4/group). **(C)** Glutamate efflux levels in the media (n = 3/group). **(D)** Intracellular cysteine levels (n = 3/group). **(E)** Intracellular ROS levels were assessed using DCFH-DA staining, measured by flow cytometry and quantified with MFI (n = 3-4/group). **(F)** MDA levels (n = 3/group). **(G)** GSH levels (n = 3/group). **(H)** Western blot analysis and quantification of ZO-1 and occludin protein expression (n = 3-4/group). Data are presented as mean ± SEM. Statistical significance was determined by Student's t-test **(A)** and 1-way ANOVA with Bonferroni post-hoc test **(B-H)**. $^{*}P < 0.05$. ns, non-significance. ov-xCT, xCT overexpression plasmid; ov-NC, negative control plasmid; DCFH-DA, 2',7'-Dichlorodihydrofluorescein diacetate.

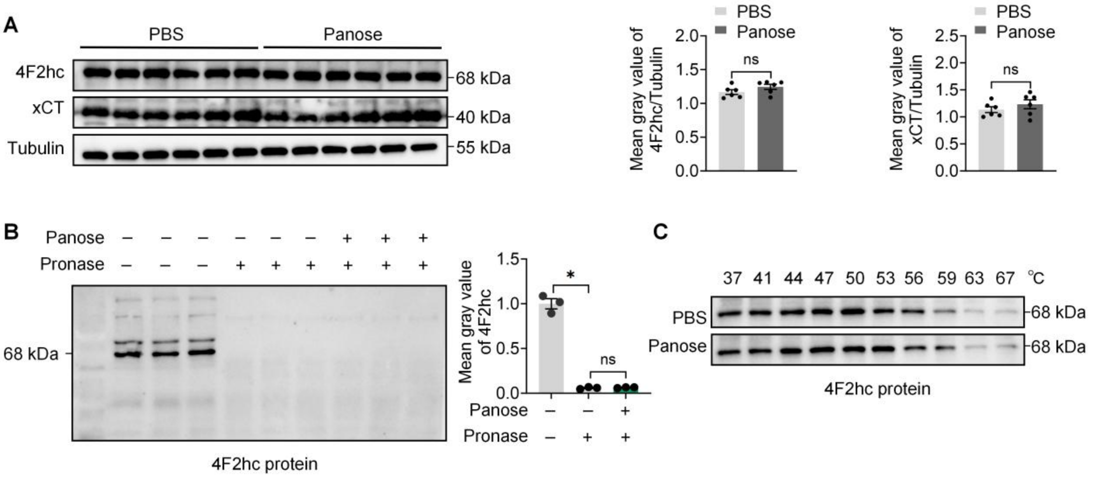

Supplementary Figure 13. Panose does not affect 4F2hc and xCT protein expression or bind to 4F2hc. (A) Western blot analysis and quantification of 4F2hc and xCT protein expression in Mode-K cells after being treated with PBS or panose for 12 h (n = 6/group). **(B)** The DARTS experiment was conducted to assess the panose-4F2hc protein interaction (n = 3/group). **(C)** The CETSA experiment was conducted to assess the panose-4F2hc protein interaction. Data are presented as mean ± SEM. Statistical significance was determined by Student's t-test **(A)** and 1-way ANOVA with Bonferroni post-hoc test **(B)**. $^{*}P < 0.05$. ns, non-significance. 4F2hc (also known as SLC3A2), solute carrier family 3 member 2; DARTS, drug affinity responsive target stability; CETSA, cellular thermal shift assays.

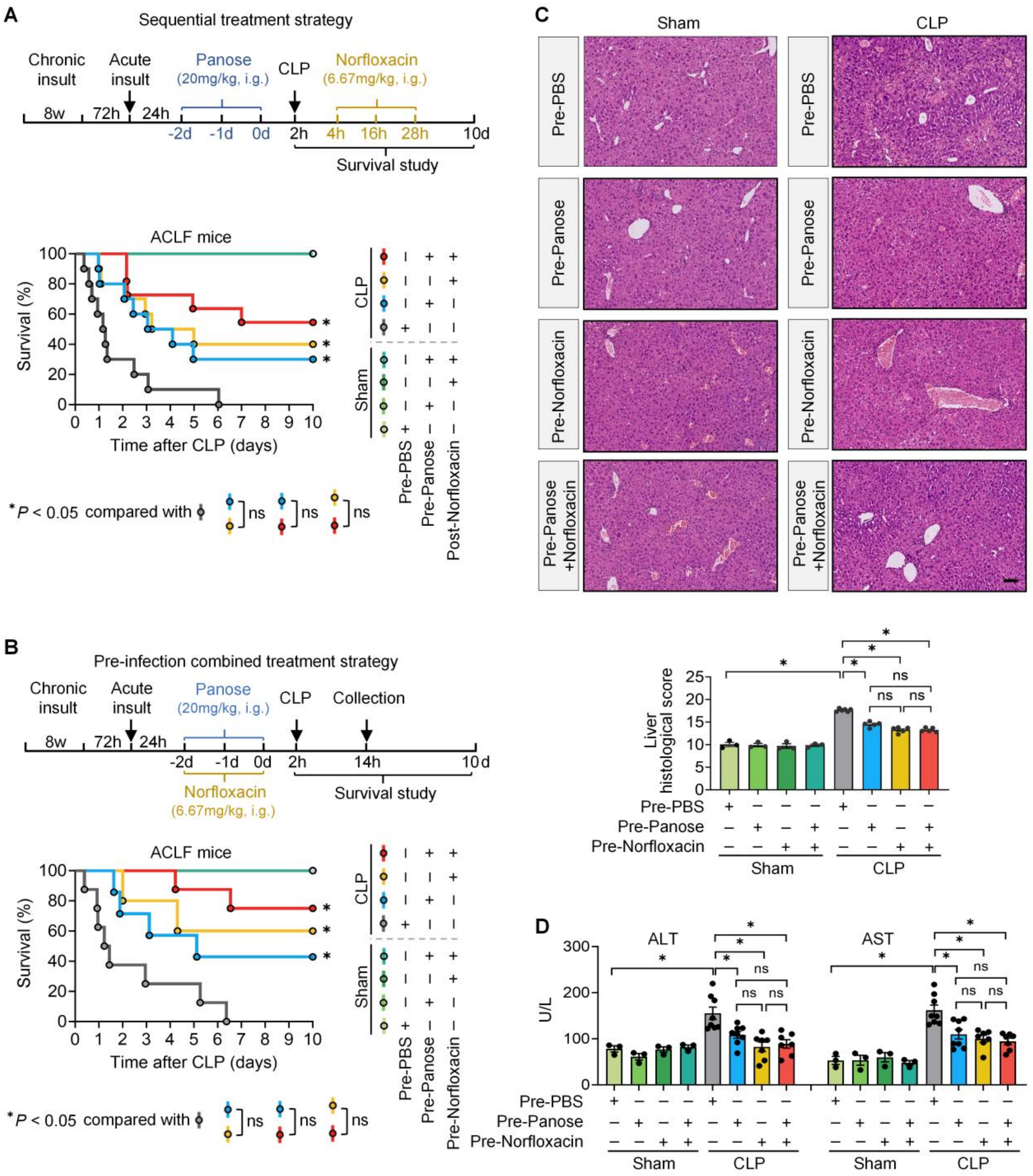

Supplementary Figure 14. Potential synergistic effects of combining panose and norfloxacin. (A) Sequential treatment strategy and survival analysis. Mice after chronic and acute insult were administered panose for 3 days before infection, followed by norfloxacin treatment at 2 h, 14 h, and 26 h post-infection. Survival outcomes were plotted using Kaplan-Meier survival curves (n = 5-11/group). **(B)** Pre-infection combined administration strategy and survival analysis. After chronic and acute insult, mice were administered panose and norfloxacin for 3 days before infection. Survival outcomes were plotted using Kaplan-Meier survival curves (n = 5-8/group). **(C)** Representative images and quantification of H&E-stained liver sections of ACLF mice that were treated with the combined administration strategy. Scale bar: 100 µm (n = 3-6/group). **(D)** Plasma levels of ALT and AST in ACLF mice that were treated with the combined administration strategy (n = 3-8/group). Data are presented as mean ± SEM. Statistical significance was determined by Log-rank test **(A and B)** and 1-way ANOVA with Bonferroni post-hoc test **(C and D)**. $^{*}P$

< 0.05. ns, non-significance.

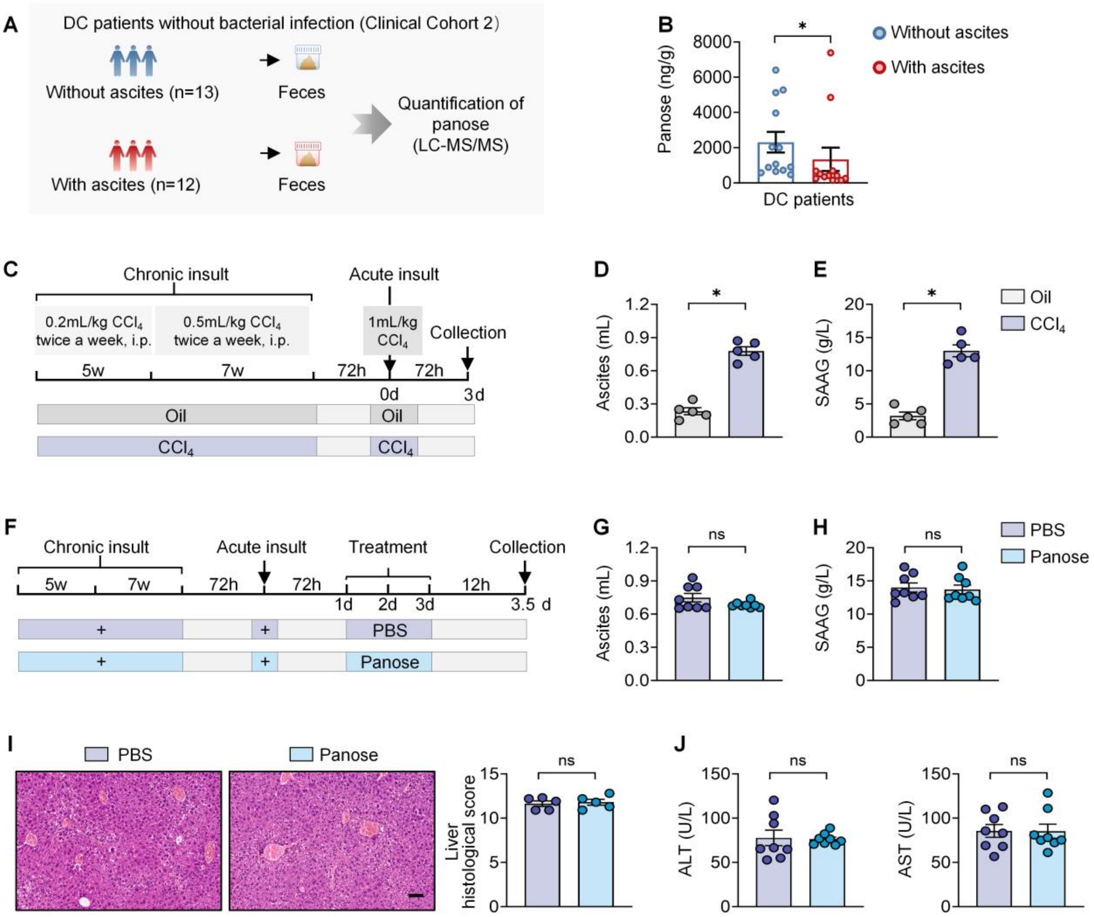

Supplementary Figure 15. Uninfected DC patients with ascites have lower panose levels, but panose administration offers no protective effect to advanced liver disease mice with ascites. **(A)** Schematic diagram of fecal sample collection in uninfected DC patients, categorized based on with or without ascites. **(B)** Fecal panose levels in uninfected DC patients with or without ascites were quantified via LC-MS/MS (n = 12-13/group). **(C)** Schematic diagram of advanced liver disease mice with ascites. Experimental mice received intraperitoneal injections of CCl_4, whereas control mice received an equal volume of olive oil. **(D)** Ascites volumes were assessed on day 3 after the acute injection (n = 5/group). **(E)** SAAG was evaluated on day 3 after the acute injection. SAAG > 11 g/L typically indicates portal hypertensive ascites (n = 5/group). **(F)** Therapeutic strategies in advanced liver disease mice with ascites. Panose was administered for 3 days starting 72 h after the acute insult, while the control mice received an equal volume of PBS. **(G)** Ascites volumes were assessed at 12 h after the final treatment (n = 8/group). **(H)** SAAG was evaluated at 12 h after the final treatment (n = 8/group). **(I)** Representative images and quantification of H&E-stained liver sections in advanced liver disease mice with ascites. Scale bar: 100 μm (n = 5/group). **(J)** Plasma ALT and AST levels in advanced liver disease mice with ascites (n = 8/group). Data are presented as mean ± SEM. Statistical significance was determined by Mann-Whitney U test **(B)** and Student's t-test **(D**, **E**, and **G-J)**. $^{*}P < 0.05$. ns, non-significance. SAAG, serum

albumin ascites gradient.

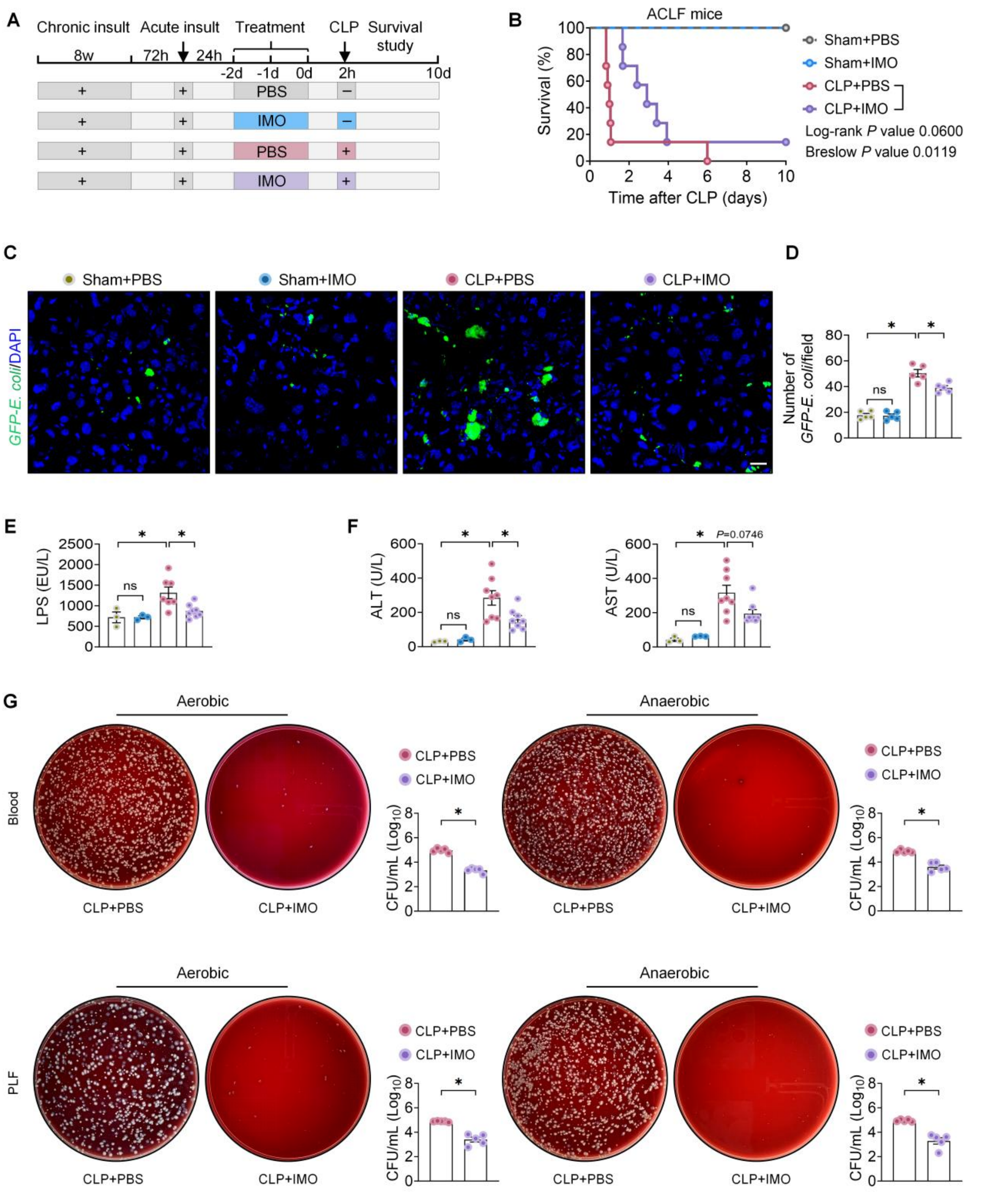

Supplementary Figure 16. IMO administration attenuates ACLF progression in mice. **(A)** Schematic timeline of IMO treatment and survival study in ACLF mice. **(B)** Kaplan-Meier survival curves of ACLF mice that were treated with IMO or an equal volume of PBS (n = 5-7/group). **(C)** Representative images of *GFP-E. coli* fluorescence intensity in the liver of ACLF mice (green: *GFP-E. coli*; blue: DAPI-stained nuclei). Scale bar: 20 µm . **(D)** Quantification of fluorescence signals of *GFP-E. coli* in the liver tissue (n = 5/group). **(E)** LPS levels in the plasma of ACLF mice at 12 h post-infection (n = 3-8/group). **(F)** Plasma levels of ALT and AST in ACLF mice at 12 h post-infection (n = 3-8/group). **(G)** Representative images and statistical plots of colony formation in peripheral blood and

PLF samples from ACLF mice after 24 h of aerobic and anaerobic incubation (n = 5/group). All CFU values were log_{10}-transformed. Data are presented as mean ± SEM. Statistical significance was determined by Log-rank test and Gehan-Breslow-Wilcoxon test **(B)**, 1-way ANOVA with Bonferroni post-hoc test **(D-F)**, and Student's t-test **(G)**. $^*P < 0.05$. ns, non-significance. IMO, isomalto-oligosaccharide.

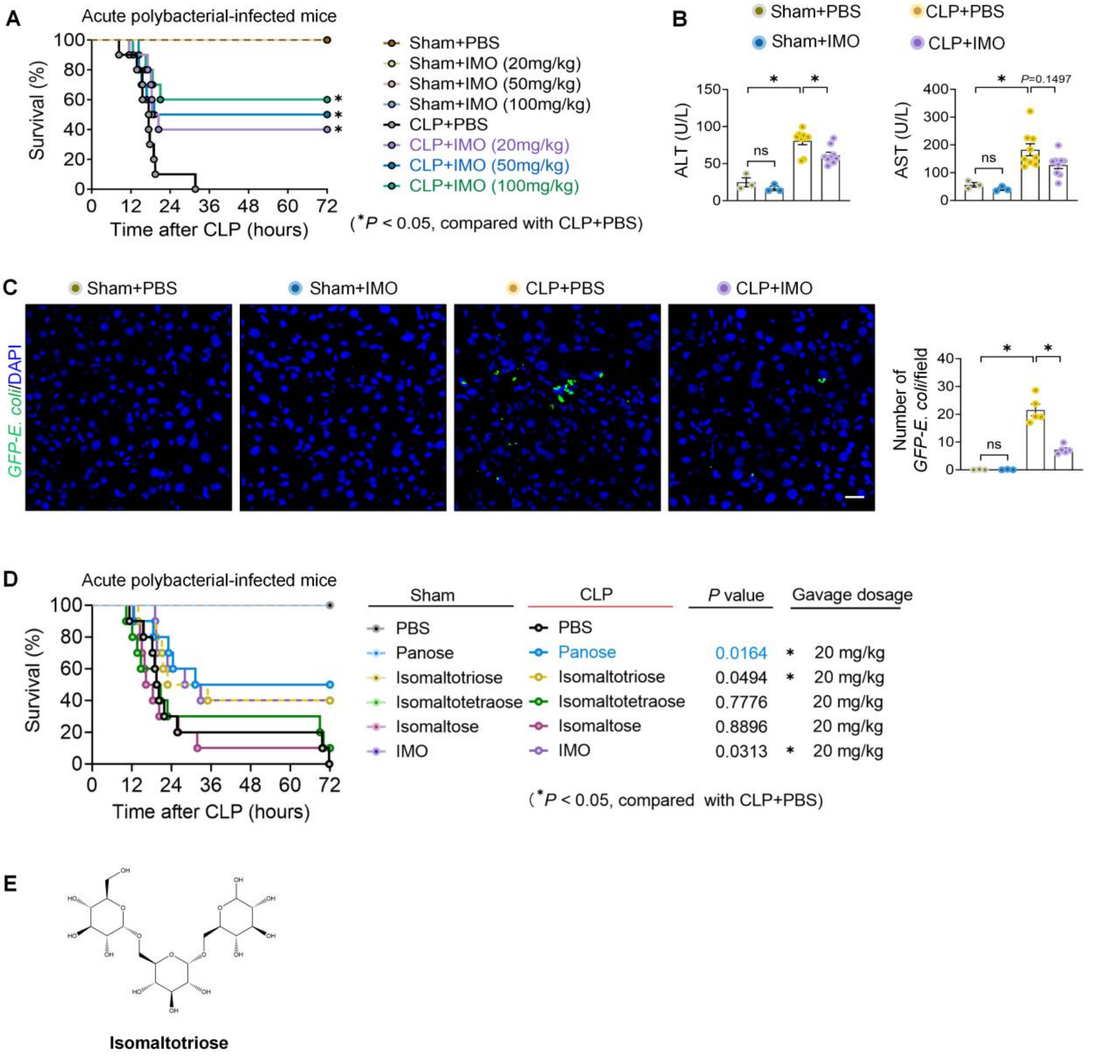

Supplementary Figure 17. IMO attenuates disease progression in acute polybacterial-infected mice, with panose being a key active component. (A) Kaplan-Meier survival curves of acute polybacterial-infected mice that were treated with different doses of IMO or an equal volume of PBS (n = 5-10/group). **(B)** Plasma levels of ALT and AST in acute polybacterial-infected mice at 12 h post-infection (n = 3-9/group). **(C)** Representative images and quantification of *GFP-E. coli* fluorescence intensity in the liver of acute polybacterial-infected mice (green: *GFP-E. coli*; blue: DAPI-stained nuclei). Scale bar: 20 μm (n = 3-5/group). **(D)** Kaplan-Meier survival curves of acute polybacterial-infected mice that were treated with PBS, panose, isomaltotriose, isomaltotetraose, isomaltose, and IMO at 20 mg/kg/day for 3 days (n = 5-10/group). **(E)** Molecular structure of isomaltotriose. Data are presented as mean ± SEM. Statistical

significance was determined by Log-rank test **(A** and **D)** and 1-way ANOVA with Bonferroni post-hoc test **(B** and **C)**. *$P < 0.05$. ns, non-significance.

Supplemental Tables

Supplementary Table 1. Demographic and clinical characteristics of uninfected and bacterial infected DC patients at admission.

**Cohort 1 related to Figure 1 A-E and Figure 2 A-F**		**Uninfected**	**Infected**
Individuals		N = 12	N = 28
Age (yr, mean ± SD)		49.00 ± 13.65	55.21 ± 11.14
Male, n (%)		10 (83.33%)	19 (67.86%)
Etiology of cirrhosis n (%)	HBV	7 (58.33%)	17 (60.71%)
	HCV	1 (8.33%)	0 (0.00%)
	HBV + AIH	0 (0.00%)	1 (3.57%)
	Alcohol	2 (16.67%)	4 (14.29%)
	Others	2 (16.67%)	6 (21.43%)
Sites of infection n (%) [@]	SBP	0 (0.00%)	8 (28.57%)
	Pneumonia	0 (0.00%)	13 (46.43%)
	Urinary tract infection	0 (0.00%)	3 (10.71%)
	Biliary tract infection	0 (0.00%)	1 (3.57%)
	Bacteremia	0 (0.00%)	3 (10.71%)
	Others	0 (0.00%)	5 (17.86%)
Indicators (mean ± SD)	ALT (U/L)	62.00 ± 64.84	150.64 ± 417.96
	AST (U/L)	71.42 ± 53.95	114.64 ± 128.75
	TBIL (μmol/L)	174.33 ± 212.64	249.54 ± 224.66
	DBIL (μmol/L)	127.43 ± 152.89	184.25 ± 159.90
	TP (g/L)	62.43 ± 7.29	57.48 ± 8.09
	ALB (g/L)	32.48 ± 5.24	30.76 ± 4.08
	CR (μmol/L)	82.50 ± 34.99	94.71 ± 39.25
	WBC ($\times 10^9$/L)	5.40 ± 2.96	7.78 ± 3.86
	LYM ($\times 10^9$/L)	1.19 ± 0.58	1.27 ± 0.62

[@] Some patients have more than one site of infection.

Supplementary Table 2. Demographic and clinical characteristics of uninfected DC patients with or without ascites at admission.

Cohort 2<br>related to Supplemental Figure 15 A and B		Without Ascites	Ascites
Individuals		N = 13	N = 12
Age (yr, mean ± SD)		55.69 ± 11.56	55.33 ± 11.32
Male, n (%)		7 (53.85%)	10 (83.33%)
Ascites (mm, mean ± SD)		0	66.33 ± 26.71
Etiology of cirrhosis n (%)	HBV	7 (53.85%)	8 (66.67%)
	Alcohol	1 (7.69%)	2 (16.67%)
	HBV + Alcohol	1 (7.69%)	0 (0.00%)
	NASH	2 (15.38%)	0 (0.00%)
	AIH	1 (7.69%)	0 (0.00%)
	Others	1 (7.69%)	2 (16.67%)
Indicators (mean ± SD)	ALT (U/L)	99.69 ± 153.44	49.83 ± 84.16
	AST (U/L)	91.38 ± 93.94	53.75 ± 33.22
	TBIL (μmol/L)	107.84 ± 139.86	62.38 ± 64.83
	DBIL (μmol/L)	76.27 ± 106.71	42.07 ± 50.65
	ALB (g/L)	34.37 ± 5.48	31.08 ± 6.04
	CR (μmol/L)	60.69 ± 13.44	102.58 ± 40.04
	WBC ($\times 10^9$/L)	4.75 ± 1.66	4.08 ± 1.85

All summary data are presented as the mean ± SD or proportions. HBV, hepatitis B virus; HCV, hepatitis C virus; NASH, nonalcoholic steatohepatitis; AIH, autoimmune hepatitis; SBP, spontaneous bacterial peritonitis; ALT, alanine aminotransferase; AST, aspartate aminotransferase; TBIL, total bilirubin; DBIL, direct bilirubin; TP, total protein; ALB, albumin; CR, creatinine; WBC, wide blood cell count; LYM, lymphocyte count.

Supplemental Table 3. Agents used in this study.

Agents	Source	Identifier
CCl_4	Macklin	C805325
Panose	Aladdin	P301896
Isomaltotriose	Aladdin	I121055
Isomaltotetraose	Aladdin	I303538
Isomaltose	Aladdin	I120961
Norfloxacin	Aladdin	N114261
Pullulan	Aladdin	P121048
Olive oil	Aladdin	O108686
N-acetylcysteine (NAC)	Selleck	S1623
PBS (vehicle)	Gibco	C10010500BT
Isomalto-oligosaccharide (IMO, food grade)	Shandong Bailong Chuangyuan Bio-Tech Co., Ltd	GB/T 20881-2017

Supplemental Table 4. Fluorescent staining antibodies.

Antibodies		Source	Identifier
Flow Cytometry Antibodies	FITC anti-Mouse CD11B antibody	Invitrogen	11-0112-82
	PE-Cyanine5 anti-Mouse F4/80 antibody	Invitrogen	15-4801-82
	APC-cy7anti-Mouse LY6G antibody	BD Biosciences	560600
Tissue Immunofluorescence Antibodies	Occludin anti-Rabbit mAb	Abcam	ab216327
	Alexa Fluor™ 488, Donkey anti-Rabbit IgG (H+L) secondary antibody	Invitrogen	R37118
	DAPI	Invitrogen	62248

Supplemental Table 5. siRNA sequences used in this study.

siRNA	Target sequence
si-xCT#1	sense, 5'-CCAGAUAUGCAUCGUCCUUTT-3'
	antisense, 5'-AAGGACGAUGCAUAUCUGGTT-3'
si-xCT#2	sense, 5'-GGAGUGCCCGGAUCCAGAUTT-3'
	antisense, 5'-AUCUGGAUCCGGGCACUCCTT-3'
si-xCT#3	sense, 5'-GCAGUCGCAGGACUGAUUUTT-3'
	antisense, 5'-AAAUCAGUCCUGCGACUGCTT-3'
si-xCT#4	sense, 5'-CCAGAAGACUCUAAAGAAUTT-3'
	antisense,5'-AUUCUUUAGAGUCUUCUGGTT-3'
si-NC	sense, 5'-UUCUCCGAACGUGUCACGUTT-3'
	antisense, 5'-ACGUGACACGUUCGGA GAATT-3'

Supplemental Table 6. Western blot antibody.

Antibodies	Source	Identifier
ZO-1 anti-Rabbit pAb	Proteintech	21773-1-AP
Occludin anti-Mouse mAb	Proteintech	66378-1-Ig
SLC7A11/xCT anti-Rabbit pAb	Proteintech	26864-1-AP
SLC3A2/4F2hc anti-Rabbit pAb	Proteintech	15193-1-AP
Alpha Tubulin anti-Rabbit pAb	Proteintech	11224-1-AP
Goat anti-Mouse IgG (H+L)-HRP	Beijing Ray Antibody Biotech	RM3001
Goat anti-Rabbit IgG (H+L)-HRP	Beijing Ray Antibody Biotech	RM3002

Supplemental Table 7. Primer sequences for selected genes.

Gene/ Bacteria	Forward (5'-3')	Reverse (5'-3')
Mus-α-Sma	GTCCCAGACATCAGGGAGTAA	TCGGATACTTCAGCGTCAGGA
Mus-Col1a1	GCTCCTCTTAGGGGCCACT	CCACGTCTCACCATTGGGG
Mus-Timp1	CCCCAGAAATCAACGAGACCA	ACCGGATATCTGCGGCATTT
Mus-Gpx-1	GACTACACCGAGATGAACGA	GGACGTACTTGAGGGAATTC
Mus-Gpx-2	GTAGTTCTCGGCTTCCCTTG	GATGCTCGTTCTGCCCATTG
Mus-Prdx-1	TTGGCGCTTCTGTGGATTCT	GGTGCGCTTGGGATCTGATA
Mus-Prdx-4	CTCCCTGCATCTAAGCAAAG	TGAGCTCCTTGAATTCTCCG
Mus-Tnf-α	CCACCACGCTCTTCTGTCTAC	AGGGTCTGGGCCATAGAACT
Mus-Il-1β	GGTCAAAGGTTTGGAAGCAG	TGTGAAATGCCACCTTTTGA
Mus-Il-6	TGATGCACTTGCAGAAAACA	ACCAGAGGAAATTTTCAATAGGC
18S	CGATCCGAGGGCCTCACTA	AGTCCCTGCCCTTTGTACACA
16S	GTGSTGCAYGGYTGTCGTCA	ACGTCRTCCMCACCTTCCTC
M. funiformis	CGGTACCTAACGAGGAAGCC	AAACCAGTTCGGACCCCATC

Supplementary Reference

1. Schloss PD, et al. Introducing mothur: open-source, platform-independent, community-supported software for describing and comparing microbial communities. *Appl Environ Microbiol.* 2009;75(23):7537-41.
2. Xiang X, et al. Interleukin-22 ameliorates acute-on-chronic liver failure by reprogramming impaired regeneration pathways in mice. *J Hepatol.* 2020;72(4):736-45.
3. Li J, et al. Gut microbial metabolite hyodeoxycholic acid targets the TLR4/MD2 complex to attenuate inflammation and protect against sepsis. *Mol Ther.* 2023;31(4):1017-32.
4. Nautiyal N, et al. Establishment of a murine model of acute-on-chronic liver failure with multi-organ dysfunction. *Hepatol Int.* 2021;15(6):1389-401.
5. Moreau R, et al. Effects of Long-term Norfloxacin Therapy in Patients With Advanced Cirrhosis. *Gastroenterology.* 2018;155(6):1816-27.e9.
6. Gu P, et al. A metabolite from commensal Candida albicans enhances the bactericidal activity of macrophages and protects against sepsis. *Cell Mol Immunol.* 2023;20(10):1156-70..
7. Krzyżewska A, et al. Cannabidiol may prevent the development of congestive hepatopathy secondary to right ventricular hypertrophy associated with pulmonary hypertension in rats. *Pharmacol Rep.* 2024;76(2):424-34.
