# Supplementary material for: Panose prevents acute-on-chronic liver failure by reducing bacterial infection in mice
Source: J Clin Invest. 2025 Jun 3;135(14):e184653. doi: 10.1172/JCI184653 (PMC12259250; doi:10.1172/JCI184653)

Unedited gel images

Fig.5B

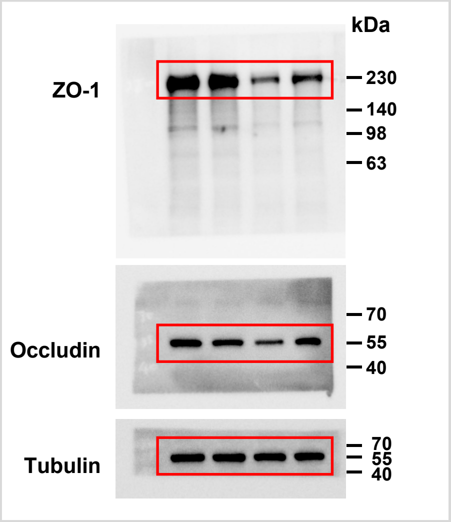

Fig.6H

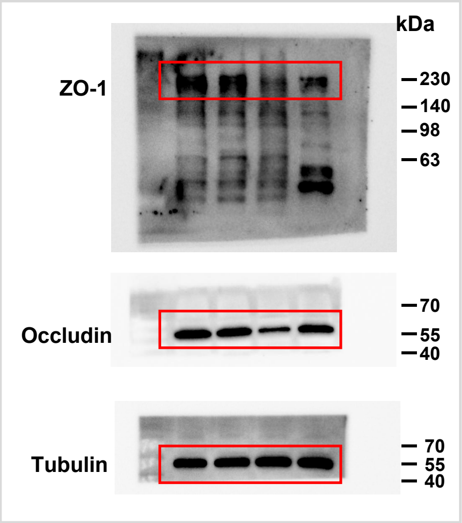

Fig. S8B

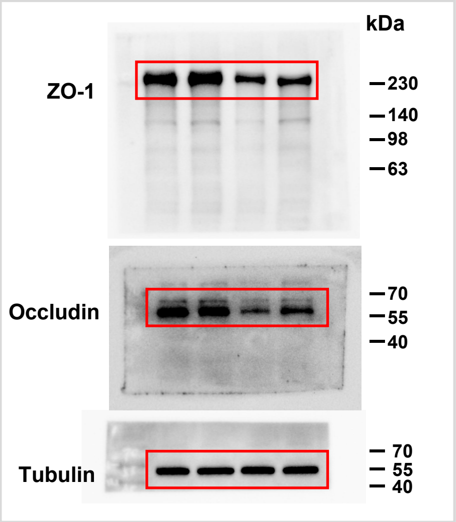

Fig.7G

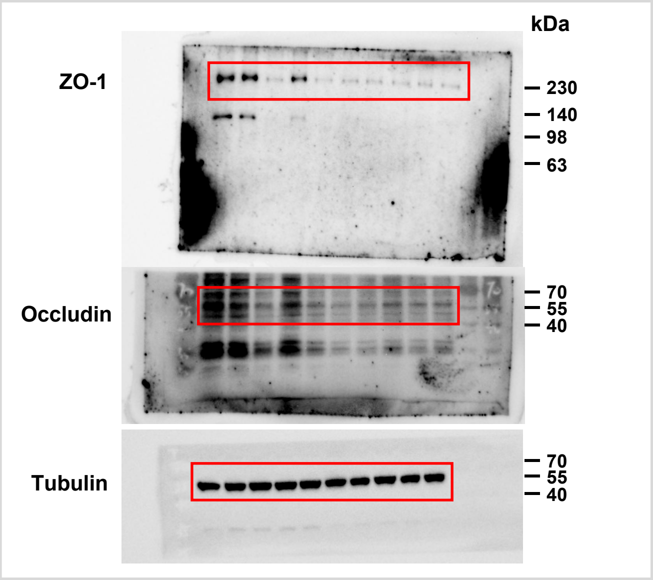

Fig.7I

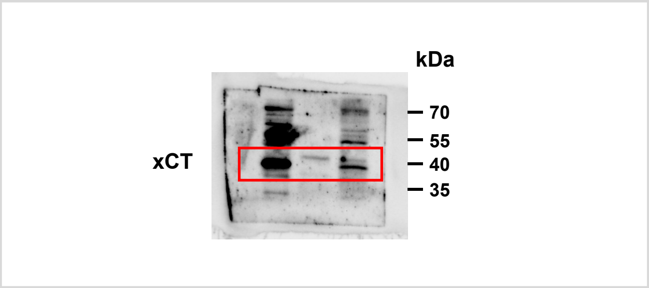

Fig.7J

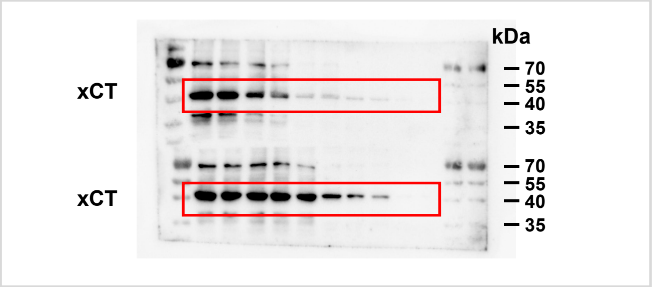

Fig.S11B

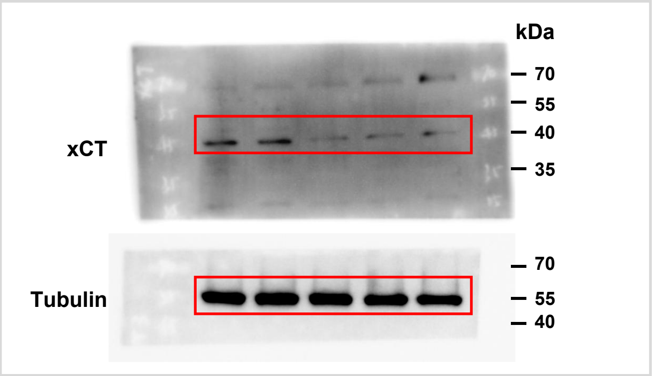

Unedited gel images

Fig.S12A

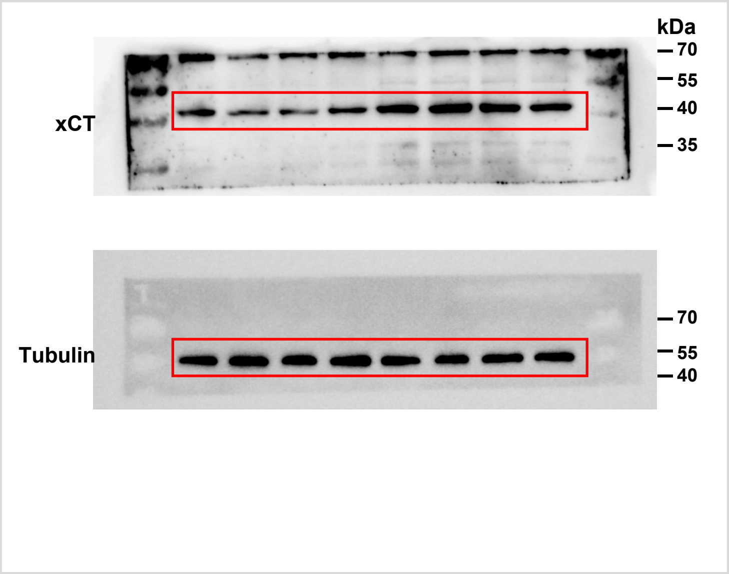

Fig.S12H

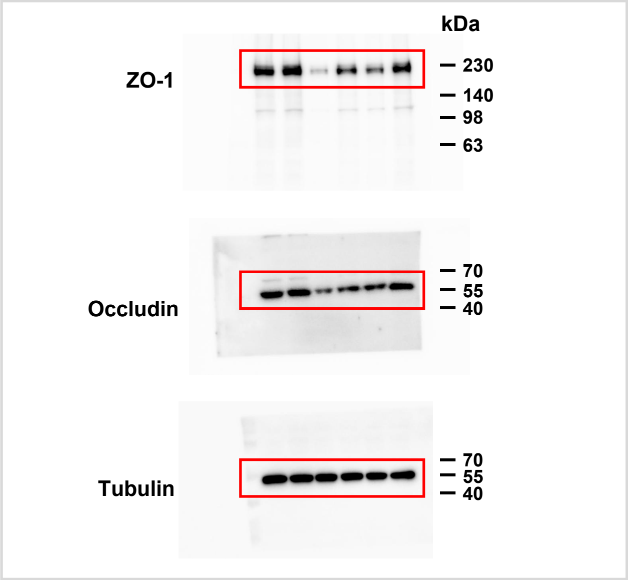

Fig.S13A

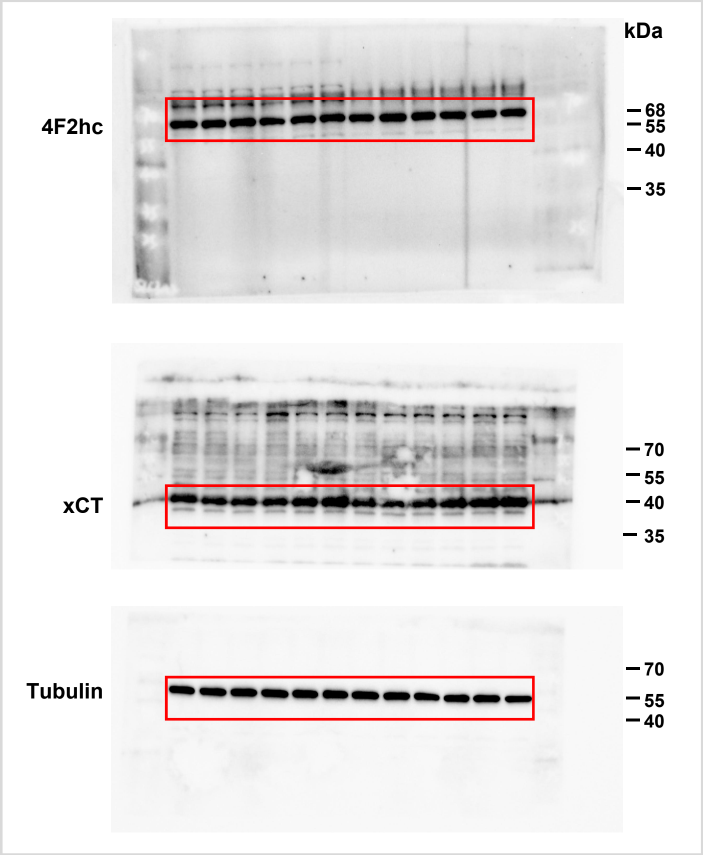

Fig.S13B

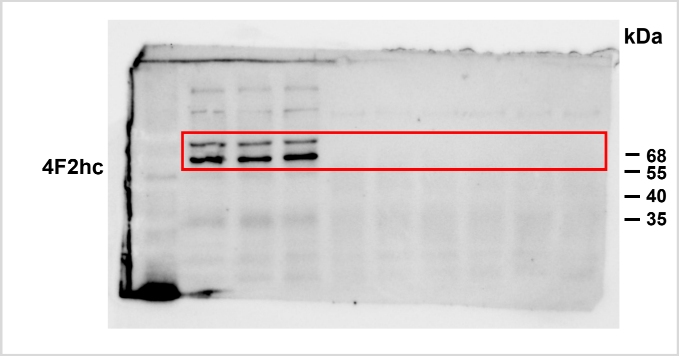

Fig.S13C

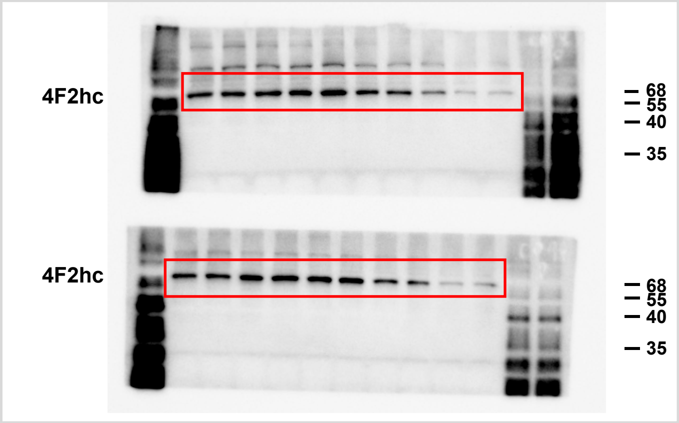

Supplement: Unedited blot and gel images [file jci-135-184653-s256.pdf]
